# Micelle-Based Ocular Inserts for Sustained Delivery and Improved Corneal Permeation of Rebamipide in Dry Eye Disease

**DOI:** 10.3390/pharmaceutics18050578

**Published:** 2026-05-07

**Authors:** Yashkumar Patel, Ketan M. Ranch, Anilkumar Prajapati, Harshilkumar Jani, Julalak Chorachoo Ontong, Sudarshan Singh

**Affiliations:** 1 Department of Pharmaceutics and Pharmaceutical Technology, L. M. College of Pharmacy, Ahmedabad 380009, Gujarat, India; yash.patel@lmcp.ac.in (Y.P.); harshil.jani@lmcp.ac.in (H.J.); 2 Research Scholar, PhD Section (Pharmacy), Gujarat Technological University, Ahmedabad 382424, Gujarat, India; anil.prajapati@lmcp.ac.in; 3 Department of Pharmacology, L. M. College of Pharmacy, Ahmedabad 380009, Gujarat, India; 4 Cosmetic Technology and Dietary Supplement Products Program, Faculty of Agro and Bio Industry, Thaksin University, Ban Pa Phayom 93210, Phatthalung, Thailand; julalak.o@tsu.ac.th; 5 High-Value Bioproduct Innovation Research Center, Faculty of Agro and Bio Industry, Thaksin University, Ban Pa Phayom 93210, Phatthalung, Thailand; 6 Office of Research Administration, Chiang Mai University, Chiang Mai 50200, Chiang Mai, Thailand; 7 Faculty of Pharmacy, Chiang Mai University, Chiang Mai 50200, Chiang Mai, Thailand

**Keywords:** rebamipide, micellar solubilization, ocular inserts, dry eye disease, non-ionic surfactants, transcorneal permeation

## Abstract

**Background:** Rebamipide (REB) is a poorly water-soluble drug with limited ocular bioavailability, necessitating advanced delivery strategies for sustained therapy in dry eye disease. **Methods:** In the present study, micelle-assisted ocular inserts were developed using non-ionic surfactants to enhance REB solubilization, drug loading, and controlled ocular delivery. The intrinsic solubility of REB in simulated tear fluid (STF, pH 7.4) was evaluated and compared with micellar systems. The formulations were characterized for particle size, polydispersity index, and zeta potential. Ocular inserts were fabricated via UV photopolymerization and evaluated for physicochemical properties, drug content, in vitro drug release, ex vivo permeation, cytocompatibility using SIRC cells, and histopathological analysis. **Results:** REB exhibited low intrinsic solubility in STF (26.05 ± 1.00 µg/mL), which was significantly enhanced in micellar systems, particularly with Solutol HS 15 (306.71 ± 1.10 µg/mL) and Tween 80 (263.18 ± 1.19 µg/mL). All micellar formulations formed stable nanosized micelles (7.5–15.1 nm) with low polydispersity (PDI < 0.35) and near-neutral zeta potential (−0.08 to −2.81 mV). The prepared ocular inserts showed uniform thickness, weight, and physiological surface pH. Micelle-assisted inserts demonstrated significantly higher drug content (87.40 ± 3.25 to 99.19 ± 2.44 µg/insert) compared to plain REB inserts (21.41 ± 2.28 µg/insert). In- vitro studies revealed sustained drug release over 24 h (92.25 ± 1.64 to 100.50 ± 1.10%), whereas plain inserts showed burst release. Ex vivo permeation studies indicated enhanced drug permeation (up to 77.30 ± 0.34 µg) and improved flux (1.38–8.52 µg/cm^2^·h) compared to plain REB. Cytocompatibility studies confirmed >90% SIRC cell viability, and histopathological analysis showed no structural damage to corneal tissue. **Conclusions:** Micelle-assisted ocular inserts, particularly those formulated with Solutol HS 15 and Tween 80, provide a promising platform for sustained, safe, and effective ocular delivery of Rebamipide in the management of dry eye disease.

## 1. Introduction

Dry eye disease (DED) is a multifactorial ocular surface disorder resulting from an imbalance in tear film homeostasis caused by inadequate tear production or excessive tear evaporation [1]. This disruption alters the delicate equilibrium among tear secretion, distribution, and drainage, leading to ocular surface damage and patient discomfort. The tear film plays a crucial role in providing lubrication, antimicrobial defense, and corneal healing support. Hence, its dysfunction compromises both ocular health and visual quality. Globally, DED affects approximately 5–30% of the population, with prevalence increasing markedly with age [2,3,4].

Pathophysiologically, DED is primarily associated with the dysfunction of the lacrimal functional unit, which includes the lacrimal glands, ocular surface, and their neural connections. A deficiency of anti-inflammatory tear components leads to the activation of T-lymphocytes, which subsequently release pro-inflammatory cytokines that induce inflammation and epithelial damage to the ocular surface [2,5,6,7]. Clinically, DED manifests through a wide spectrum of symptoms such as ocular pain, redness, burning sensation, excessive tearing, foreign body sensation, and fluctuating vision. These symptoms not only cause physical discomfort but also significantly reduce quality of life, impairing daily activities like reading, driving, and extended screen use. Such widespread impact emphasizes the necessity of developing effective, patient-compliant therapeutic options for managing DED [8,9,10,11].

The goals of DED treatment are to restore the ocular surface and normalize tear film production, improve patient comfort, and alleviate symptoms, with current therapeutic approaches ranging from artificial tear formulations, anti-inflammatory agents, mucin secretagogues, corticosteroids, dietary supplements such as omega-3 fatty acids, vitamin A, autologous serum eye drops, antibiotics including tetracyclines and macrolides, immunomodulators such as tacrolimus, punctal plugs, and surgical interventions [12,13]. Among these options, rebamipide (REB) offers a unique therapeutic advantage by directly addressing mucin deficiency, a key pathological factor in DED, thereby restoring tear film stability and promoting ocular surface healing rather than providing only symptomatic relief [14,15]. REB, a quinolinone derivative, was originally developed as a mucosal protective agent for treating gastric ulcers and chronic gastritis due to its potent mucin-enhancing and anti-inflammatory properties [16]. It stimulates mucin secretion in gastric epithelial cells, scavenges reactive oxygen species, and suppresses inflammatory cytokine production, thereby supporting mucosal healing and barrier integrity [17]. Beyond gastrointestinal applications, REB has gained considerable attention in ophthalmology, particularly for DED management, where it functions as a mucin secretagogue that restores tear film stability and promotes epithelial healing. Clinical and preclinical studies have demonstrated that REB increases the number of conjunctival goblet cells and promotes secretion of mucin-like glycoproteins in both corneal and conjunctival epithelia, improving lubrication and overall ocular surface integrity [2,5].

The ophthalmic suspension of REB, commercially available as Mucosta^®^ Ophthalmic Suspension UD 2% by Otsuka Pharmaceutical Co. (Japan), was approved in 2011 following successful clinical trials. Patients treated with four daily doses of this formulation showed significant improvements in tear film breakup time, vital staining scores, and overall ocular comfort, confirming REB’s therapeutic efficacy in DED [5]. Mechanistic studies have revealed that REB upregulates the expression of membrane-associated mucins such as MUC1, MUC4, and MUC16 through activation of the epidermal growth factor receptor signaling pathway, thereby enhancing epithelial barrier protection and maintaining ocular hydration [18]. Additionally, REB suppresses T-cell activation and cytokine release, contributing to reduced ocular inflammation and accelerated corneal wound healing.

Despite its therapeutic advantages, several formulation-related challenges restrict the clinical efficacy of REB. The drug is practically insoluble in water and exhibits very low solubility in pH-neutral buffer systems appropriate for ocular use, leading to poor bioavailability [19]. The marketed suspension (Mucosta^®^ 2%) is a milky, turbid formulation requiring vigorous shaking before use to re-disperse settled particles. This opacity can cause transient blurred vision and ocular irritation due to its high drug concentration (20 mg/mL), which negatively impacts patient compliance [16]. Furthermore, REB is categorized as a Biopharmaceutical Classification System (BCS) Class IV drug, characterized by both low solubility and low permeability, with logP and pKa values of 2.9 and 3.3, respectively. Consequently, only a limited fraction of the topically administered dose remains on the ocular surface before being eliminated through tear turnover and nasolacrimal drainage, necessitating frequent dosing to maintain therapeutic levels [20]. Additionally, manufacturing sterile ophthalmic suspensions of REB poses technical challenges, as standard 0.2 µm filtration methods cannot be used for sterilization, complicating large-scale production and increasing manufacturing costs [19]. These limitations collectively underscore the need for advanced delivery systems capable of improving REB solubility, stability, bioavailability, and patient adherence.

Recent research has focused on innovative drug delivery strategies to overcome the inherent limitations of REB. Liposomal REB formulations have demonstrated improved ocular retention, reduced irritation, and sustained release, offering superior therapeutic performance compared to conventional suspensions [16]. Similarly, nanoparticle-based sustained-release systems, developed using bead milling with 2-hydroxypropyl-β-cyclodextrin and methylcellulose, achieved particle sizes between 40 and 200 nm and showed enhanced corneal penetration, sustained mucin secretion, and tear film stabilization in rabbit models [20]. Another approach involved developing clear, supersaturated aqueous eye drops using pH modification and hydrophilic polymers such as hydroxypropyl methylcellulose (4.5 cp). These optimized formulations demonstrated improved solubility, stability, and bioavailability, while minimizing dosing frequency and visual disturbance [19]. Moreover, cationic β-cyclodextrin copolymers significantly enhanced REB solubility and corneal retention without inducing toxicity, improving mucin secretion and overall therapeutic efficacy in experimental DED models [21]. Collectively, these findings highlight the promising evolution of REB delivery platforms liposomes, nanoparticles, supersaturated solutions, and β-cyclodextrin complexes that enhance its ocular bioavailability and patient comfort.

Nevertheless, these systems still face limitations in achieving long-term retention and controlled release on the ocular surface. REB’s poor aqueous solubility and rapid precorneal elimination continue to hinder sustained therapeutic exposure [22]. To address these shortcomings, micellar systems have emerged as an effective nanocarrier platform for ocular drug delivery. Micelles are self-assembled colloidal structures formed by amphiphilic polymers above their critical micelle concentration (CMC). They consist of a hydrophobic core that solubilizes poorly water-soluble drugs and a hydrophilic shell that stabilizes the system in an aqueous environment. Incorporating REB into polymeric micelles not only enhances its solubility and stability but also facilitates trans-corneal permeation and cellular uptake. Building on this concept, the present research utilizes a micellar–ocular insert hybrid system for the delivery of REB. In this approach, a micellar solution containing solubilized REB is used to impregnate pre-formed polymeric ocular inserts, enabling efficient drug loading through diffusion and adsorption within the polymeric matrix. Upon administration, these micelle-laden inserts provide sustained drug release and enhanced residence time compared to conventional eye drops. Unlike plain REB solution, which is rapidly cleared from the ocular surface, the insert ensures continuous and localized delivery, thereby improving therapeutic efficacy and patient compliance [23,24]. Such a combination strategy integrates the solubilization advantage of micelles with the prolonged retention properties of ocular inserts, creating a synergistic platform that can overcome multiple formulation challenges simultaneously. The micellar ocular insert is therefore proposed as a novel, patient-friendly, and efficient delivery system for enhanced management of dry eye disease [25,26].

## 2. Materials and Methods

Rebamipide (REB; Mw: 370.35 g·mol^−1^, purity ≥ 98%) was provided as a gift sample by Dr. Reddy’s Laboratories Ltd., Hyderabad, India. Pluronic^®^ F68 (Poloxamer 188; average Mw ~8400 g·mol^−1^) and Pluronic^®^ F127 (Poloxamer 407; average Mw ~12,600 g·mol^−1^) were purchased from Sigma-Aldrich Chemicals Pvt. Ltd., Bangalore, India. Polysorbate 20 (Tween^®^ 20; average Mw ~1227 g·mol^−1^) and polysorbate 80 (Tween^®^ 80; average Mw ~1310 g·mol^−1^) were obtained from Merck Life Science Pvt. Ltd., Mumbai, India. Caprylocaproyl polyoxyl-8 glycerides (Labrasol^®^; average Mw ~365 g·mol^−1^) and polyoxyethylene esters of 12-hydroxystearic acid (Solutol^®^ HS 15; average Mw ~963 g·mol^−1^) were procured from Gattefossé India Pvt. Ltd., Mumbai, India. Sodium chloride (NaCl; Mw: 58.44 g·mol^−1^), potassium chloride (KCl; Mw: 74.55 g·mol^−1^), sodium bicarbonate (NaHCO_3_; Mw: 84.01 g·mol^−1^), and calcium chloride dihydrate (CaCl_2_·2H_2_O; Mw: 147.02 g·mol^−1^) were obtained from Finar Chemicals Ltd., Ahmedabad, India. Fluid thioglycollate medium and soybean–casein digest medium were purchased from HiMedia Laboratories Pvt. Ltd., Mumbai, India. All chemicals and excipients used were of pharmaceutical or analytical grade. Ultrapure water was used throughout the experimental work.

### 2.1. Micelle-Assisted Solubilization Study of REB

**Figure 1:**
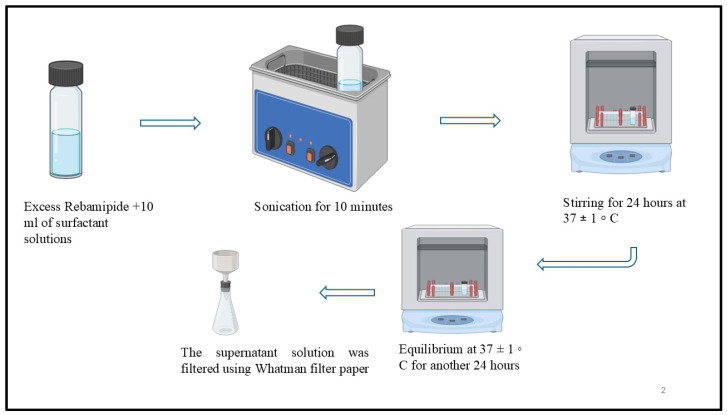
Micelle-assisted solubilization of REB.

A micelle-assisted solubilization study was performed to evaluate the affinity of REB toward various surfactants (Figure 1) and to identify the most suitable surfactant for achieving maximum drug loading in micellar drug delivery systems. For determining the micellar solubilization capacity, an excess amount of REB was added to 10 mL of aqueous surfactant solutions prepared at concentrations equivalent to ten times their respective CMC, namely Pluronic F127 (0.5% *w*/*v*), Pluronic F68 (0.5% *w*/*v*), Tween 80 (0.015% *w*/*v*), Tween 20 (0.06% *w*/*v*), Solutol HS 15 (0.2% *w*/*v*), and Labrasol (0.042% *w*/*v*). Maintaining the surfactant concentration above the CMC ensured stable and complete micellization. The experiments were carried out in 40 mL glass vials. The drug–surfactant mixtures were initially sonicated for 10 min at room temperature to enhance drug dispersion within the micellar phase. Subsequently, the samples were subjected to continuous stirring in an orbital shaking incubator at 37 ± 1 °C for 24 h to facilitate drug incorporation into the micelles. After stirring, the mixtures were allowed to stand undisturbed at 37 ± 1 °C for an additional 24 h to attain thermodynamic equilibrium. Upon completion of the equilibration period, the samples were filtered through Whatman Grade 1 filter paper to remove undissolved drug. The clear filtrates were then analyzed using a UV–Visible spectrophotometer to quantify the concentration of REB solubilized within the micellar systems. The use of UV–Visible spectroscopy for quantification in micellar and colloidal systems is well supported in the literature [27,28].

### 2.2. Stability During Dilution

This study evaluated the dilution-induced stability of REB-loaded micelles under conditions mimicking ocular insert wear. During wear, micelles are continuously exposed to tear fluid, resulting in progressive dilution. As the surfactant concentration decreases below the CMC, micelle destabilization may occur, potentially causing premature drug release and a subsequent burst effect. Thus, it was hypothesized that if micelle destabilization took place upon dilution, the released REB would precipitate, leading to a decrease in transmittance (increased turbidity) due to drug precipitates. To assess this, the stability of REB-loaded micelles in the presence of tear fluid was investigated using different surfactant-based formulations. Each formulation was diluted 10-fold and 100-fold with simulated tear fluid (STF; 0.9% *w*/*v* NaCl and 0.015% *w*/*v* NaHCO_3_, pH-7.4). The percentage transmittance of the diluted samples was measured at 480 nm using UV–visible spectrophotometry (Shimadzu UV-1900, Kyoto, Japan).

### 2.3. Size, Polydispersity Index, and Zeta Potential Analysis

The size, polydispersity index (PDI), and zeta potential (ZP) of the REB-loaded micellar formulations were characterized using dynamic light scattering (DLS) and electrophoretic light scattering techniques employing a Malvern Zetasizer Nano ZS-90 (Malvern Instruments, Malvern, UK). Particle size and PDI were determined by transferring approximately 1 mL of the sample into a disposable polystyrene cuvette (Malvern Panalytical). Measurements were carried out at 25 ± 0.5 °C at a fixed scattering angle of 90°, using automatic attenuation settings. The PDI values were used to assess the homogeneity and size distribution of the micellar systems, with lower PDI values indicating narrow size distributions and minimal aggregation. Zeta potential measurements were carried out to evaluate the surface charge and colloidal stability of the micelles. For this purpose, approximately 1 mL of the sample was loaded into a folded capillary zeta cell. with a flat-bottom configuration. Measurements were conducted at 25 ± 0.5 °C, and the electrophoretic mobility was recorded using electrophoretic light scattering.

### 2.4. Fabrication of Ocular Inserts

Ocular inserts were prepared using a UV-curable monomer system through a photopolymerization process. The prepolymer formulation consisted of Irgacure D (10 mg) as the photo-initiator, ethylene glycol dimethacrylate (EGDMA, 5 µL) as the crosslinking agent, N,N-dimethylacrylamide (DMA, 310 µL), N-vinylpyrrolidone (NVP, 15 µL), a siloxane macromer (100 µL), and 2-hydroxyethyl methacrylate (HEMA), which was added to adjust the final volume to 1 mL. All components were accurately measured and transferred into an amber glass vial to prevent premature photoactivation, followed by gentle mixing to obtain a clear and homogeneous prepolymer solution. The formulation was then degassed by bath sonication for 10 min at room temperature to eliminate entrapped air and dissolved gases. A predetermined volume of the degassed prepolymer solution was dispensed onto a precleaned glass slide, and a second glass slide was carefully placed over it to form a uniform thin film. The assembled slides were exposed to ultraviolet irradiation at 365 nm for 5 min to achieve complete photopolymerization. After curing, the polymerized film was carefully removed from the glass substrate. Circular ocular inserts of uniform dimensions were punched using a sterile stainless-steel borer. To remove any residual unreacted monomers, the inserts were immersed in deionized water and boiled for 15 min. The purified inserts were dried by air-drying at room temperature (25 ± 2 °C) and subsequently stored in sterile, airtight containers until further evaluation.

### 2.5. Micelle-Assisted Passive Loading of REB into Ocular Inserts

REB was loaded into the fabricated ocular inserts using a post-fabrication passive diffusion technique. For micelle-assisted loading, the inserts were completely immersed in a pre-optimized REB-loaded micellar solution and incubated in an orbital shaking incubator at 37 ± 1 °C with continuous agitation for 7 days to facilitate diffusion-driven drug uptake into the polymeric matrix. To assess the influence of micellar solubilization on loading efficiency, a control group consisting of plain REB-loaded inserts (micelle-free) was prepared using a conventional aqueous loading approach. In this method, the inserts were immersed in a saturated REB solution prepared in STF and incubated under identical conditions of temperature and agitation for 7 days. Upon completion of the loading period, the inserts were removed and gently rinsed with distilled water to eliminate any surface-associated drug. The inserts were then blotted dry using lint-free tissue, dried under controlled conditions, and stored in sterile containers until further physicochemical characterization and performance evaluation.

### 2.6. Sterilization and Sterility Testing

The fabricated ocular inserts were sterilized by moist heat sterilization using an autoclave (Equitron, SLE Series, Mumbai, India). Briefly, individual inserts were placed in sterile glass vials containing STF to maintain hydration and prevent structural deformation during sterilization. The vials were loosely capped and subjected to autoclave at 121 °C under a pressure of 15 psi for 20 min. After completion of the sterilization cycle, the vials were allowed to cool gradually to room temperature and were subsequently sealed under aseptic conditions. Sterility testing of the autoclaved ocular inserts was performed to detect the presence of viable microorganisms using the direct inoculation method, in accordance with pharmacopeial guidelines. Fluid thioglycollate medium and soybean–casein digest medium was used to support the growth of anaerobic and aerobic microorganisms, respectively. The culture media were prepared as per standard procedures, and 20 mL of each medium was transferred into sterile test tubes, which were cotton-plugged and sterilized by autoclaving at 121 °C for 20 min. Under aseptic conditions, sterilized ocular inserts were directly inoculated into test tubes containing the respective media. The tubes containing fluid thioglycollate medium were incubated at 30–35 °C, while those containing soybean–casein digest medium were incubated at 20–25 °C. All samples were observed periodically for a duration of 14 days for any evidence of microbial growth, as indicated by turbidity or visible sediment. Un-inoculated media served as negative controls to confirm media sterility.

### 2.7. Physicochemical Evaluation of Ocular Inserts

#### 2.7.1. Appearance, Thickness, Weight Uniformity, Surface pH

The physical appearance of all fabricated inserts was visually inspected for surface uniformity, transparency, and the absence of particulate matter or air bubbles. The thickness of each insert was measured at three different positions using a digital micrometer (EIE ACCUPLUS Instruments Digital Micrometer, EIE Instruments Pvt. Ltd., Ahmedabad, India). The ocular inserts were individually weighed using an analytical balance (Mettler Toledo AG245, Greifensee, Switzerland) to assess weight uniformity.

The surface pH of the ocular inserts was determined to evaluate their compatibility with the ocular surface. Briefly, each insert was placed in a closed Petri dish containing 1 mL of distilled water and allowed to swell at room temperature for 30 min. After swelling, the insert was removed, and the remaining solution was gently stirred. The surface pH was then measured using a calibrated digital pH meter (Systronics Digital pH Meter 335, Systronics India Ltd., Ahmedabad, Gujarat, India). All measurements were performed carefully to ensure stable readings.

#### 2.7.2. Moisture Absorption and Moisture Loss

Moisture uptake and loss were assessed to evaluate the hygroscopic behavior and stability of the ocular inserts. In brief, three inserts of equal size were selected from each batch, and their initial individual weights were recorded. For the moisture absorption study, the inserts were placed in a desiccator containing a saturated sodium chloride (NaCl) solution, which maintains a controlled relative humidity of approximately 75% [29]. The desiccator was sealed securely and kept undisturbed for three days to allow the samples to equilibrate. At the end of the exposure period, the inserts were removed, gently blotted to remove any surface moisture, and reweighed. The percentage moisture absorption was then calculated using the following equation.

$$\mathrm{Moisture}\;\mathrm{absorption}\;(\%)=\frac{\mathrm{Final}\;\mathrm{weight}-\mathrm{Initial}\;\mathrm{weight}}{\mathrm{Initial}\;\mathrm{weight}}\times 100$$

Furthermore, to determine moisture loss, fresh insert samples were placed in a separate desiccator containing anhydrous calcium chloride (5 g), which develop a low-humidity environment (RH ≈ 17%). The desiccator was sealed and maintained for three days to allow the inserts to equilibrate. After this period, the inserts were carefully removed and reweighed. The percentage moisture loss was then calculated using the following equation.

$$\mathrm{Moisture}\;\mathrm{loss}\;(\%)=\frac{\mathrm{Initial}\;\mathrm{weight}-\mathrm{Final}\;\mathrm{weight}}{\mathrm{Initial}\;\mathrm{weight}}\times 100$$

#### 2.7.3. Swelling Index

The swelling behavior of the ocular inserts was assessed in STF to mimic ocular conditions. The dry weight of each insert was accurately recorded before the experiment. Each insert was then placed in a Petri dish containing 5 mL of STF and allowed to hydrate. At predetermined time intervals, the inserts were carefully removed, and excess surface fluid was gently blotted with filter paper to avoid compression. The inserts were then immediately reweighed. The swelling index (%) was calculated using the following formula:

$$\mathrm{Swelling}\;\mathrm{index}\;(\%)=\frac{\mathrm{Weight}\;\mathrm{of}\;\mathrm{swollen}\;\mathrm{insert}-\mathrm{Initial}\;\mathrm{weight}}{\mathrm{Initial}\;\mathrm{weight}}\times 100$$

#### 2.7.4. Folding Endurance and In Vitro Degradation Study

The flexibility and mechanical integrity of the prepared ocular inserts were evaluated using the folding endurance test. A specified region of each insert (2.0 cm^2^) was repeatedly folded at the same point until either a visible break occurred or the insert was folded up to 300 times, which was set as the end point. The total number of folds the insert could withstand without breaking was recorded as the folding endurance value. This parameter provides an indication of the mechanical strength and handling resilience of the inserts during storage and application. The ocular inserts were incubated in STF at 37 °C for 7 days. At predetermined time points, the pH of the incubation medium was measured using a digital pH meter (Systronics 335, Systronics India Ltd., Ahmedabad, Gujarat, India) to monitor changes associated with degradation.

#### 2.7.5. Uniformity of Drug Content

The uniformity of drug distribution in the ocular inserts was assessed by randomly selecting three inserts from each formulation. Each insert was placed in a glass vial containing 10 mL of methanol and dissolved under continuous agitation using an orbital shaker (Remi CIS 18 plus, Remi Elektrotechnik Ltd., Mumbai, India). The solution was then filtered to remove any undissolved excipients. A 3 mL aliquot of the filtrate was withdrawn, and the absorbance was measured at 228 nm using a UV–visible spectrophotometer.

#### 2.7.6. In Vitro Release Studies

The in vitro release of the drug from the ocular inserts was evaluated using STF. Each sterile ocular insert was placed in a glass vial containing 2 mL of STF and incubated at 32 ± 1 °C in an orbital shaker incubator (CIS-18 Plus, REMI Elektrotechnik Ltd., Mumbai, India) at 100 rpm. This setup was designed to simulate in vivo tear turnover while keeping the dissolution medium volume constant. At predetermined time intervals, 2 mL of STF was withdrawn to measure drug release, and an equal volume of fresh STF was immediately added to maintain sink conditions. The drug concentration was determined using UV–visible spectroscopy, and the cumulative drug release (%) was plotted over time to construct the release profile.

#### 2.7.7. Drug Release Kinetics and Mechanistic Modeling

The release behavior of the formulated ocular inserts was examined using several mathematical models to identify the dominant mechanism governing drug diffusion from the polymer matrix. The experimental release data were fitted to zero-order, first-order, Higuchi, Hixson–Crowell, and Korsmeyer–Peppas models, as each provides insight into different aspects of drug transport. Zero-order and first-order models were used to evaluate whether the drug was released at a constant rate or whether release depended on declining concentration. The Higuchi model was included to assess Fickian diffusion from the polymer network, while the Hixson–Crowell model helped determine if changes in geometry or surface area contributed to release. The Korsmeyer–Peppas model was particularly useful for distinguishing between pure diffusion, anomalous behaviour, or polymer-relaxation-driven mechanisms based on the diffusion exponent (n). For each model, the linear regression coefficient (R^2^) and kinetic constants were calculated, and the best-fit model was identified by comparing R^2^ values and the behaviour of the diffusion exponent. This combined kinetic evaluation enabled a mechanistic understanding of how the inserts-controlled drug release and clarified whether diffusion, erosion, or a combination of both contributed to the observed release pattern.

#### 2.7.8. Ex Vivo Transcorneal Permeation

The ex vivo permeation behavior of the developed ocular inserts was assessed using Franz diffusion cell to simulate transcorneal drug transport. Fresh corneas were carefully excised from got eyes and immediately rinsed with cold isotonic saline to remove residual proteins. Each cornea was positioned between the donor and receptor chambers of the Franz cell, ensuring that the epithelial surface faced the donor compartment. The receptor chamber was filled with STF (pH 7.4), and the system temperature was maintained at 34 ± 0.5 °C using a circulating water jacket. Gentle magnetic stirring facilitated mixing throughout the experiment. The donor chamber received the test insert or control solution, and receptor samples were collected at predetermined intervals. Each withdrawn aliquot was immediately replaced with an equal volume of fresh buffer to preserve sink conditions. The samples were analyzed using the validated uv visible spectroscopy method, and the cumulative amount permeated (Q_n_) at each time point was calculated using:

$$Q_{n}=V_{r}C_{r}(n)+\sum_{x=1}^{n}V_{s}C_{r}(x-1)$$

where $V_{r}$ is the receptor volume, $C_{r}(n)$ is the drug concentration at sampling time n, and $V_{s}$ is the withdrawn sample volume. The steady-state flux (Jss) was 0 to:

$$J_{ss}=\frac{dQ/dt}{A}$$

where A represents the corneal diffusion area.

### 2.8. Cytocompatibility Assay

The cytocompatibility of the fabricated ocular inserts was assessed using Statens Seruminstitut Rabbit Cornea (SIRC) cells procured from the National Centre for Cell Science (NCCS), Pune, Maharashtra, India, by evaluating cell viability through the MTT assay. Ocular insert extracts were prepared by incubating individual inserts in 2 mL of simulated tear fluid (STF) at 34 °C under continuous agitation at 100 rpm for 24 h using an orbital shaker (CIS-18 Plus, REMI Elektrotechnik Ltd., Mumbai, India). SIRC cells were seeded into sterile 96-well culture plates at a density of 2 × 10^3^ cells per well and incubated for 24 h to allow cell attachment. Subsequently, the culture medium was replaced with the prepared ocular insert extracts, and the cells were further incubated for 24 h. After the treatment period, MTT solution (5 mg/mL) was added to each well and the plates were incubated at 37 °C for 2 h to facilitate the formation of formazan crystals. The culture medium was then carefully removed without disturbing the crystals, and 150 µL of dimethyl sulfoxide (DMSO) was added to each well to dissolve the formazan. The absorbance was measured at 540 nm using a Varioskan Flash ELISA plate reader (Thermo Scientific, Waltham, MA, USA). Cell viability was calculated and expressed as a percentage relative to the untreated control group.

### 2.9. Histopathological Evaluation

Fresh goat eyes were procured from a local slaughterhouse and transported under sterile, cooled conditions for immediate processing. The corneas were carefully excised and randomly allocated into three experimental groups negative control, positive control, and treatment group. The negative control group was exposed to simulated tear fluid to represent physiological conditions, while the positive control group was treated with absolute alcohol to induce corneal damage. The treatment group was exposed to the developed test formulation. All samples were incubated under controlled conditions for 24 h. Following the exposure period, the corneal tissues were subjected to histopathological evaluation. The tissues were fixed in an appropriate fixative, dehydrated through a graded alcohol series, and embedded in paraffin. Sections of suitable thickness were prepared using a microtome and stained with hematoxylin and eosin (H&E) for microscopic analysis. The stained sections were examined using an inverted microscope (Olympus, Tokyo, Japan) equipped with a QImaging Micropublisher 3.3 RTV camera and operated via QCapture Pro software (version 7). The evaluation focused on assessing the structural integrity of the corneal layers, including the epithelium, stroma, and endothelium, as well as identifying any morphological alterations or signs of tissue damage induced by the treatment.

### 2.10. Statistical Analysis

Statistical analysis was performed using one way ANOVA to compare the results obtained among tested components using GraphPad Prism (version 10.5). All experiments were performed in triplicate and presented as mean ± standard deviation.

## 3. Results and Discussion

### 3.1. Micelle-Assisted Solubilization Study of REB

The solubility study was carried out to select the most suitable surfactant for enhancing the micellar solubilization of REB (Table S5), a highly hydrophobic and poorly water-soluble drug. The intrinsic solubility of REB in STF (pH 7.4) was 26.05 ± 1.00 µg/mL, confirming its limited solubility in aqueous media. All tested surfactants significantly improved REB solubility, compared to water clearly indicates that micelle formation at higher CMC levels facilitates the incorporation of REB into their hydrophobic cores. Among the surfactants tested, Solutol^®^ HS 15 showed the highest solubilization capacity (306.71 ± 1.10 µg/mL), followed by Tween^®^ 80 (263.18 ± 1.19 µg/mL). Pluronic^®^ F68, although still improving solubility relative to STF, showed the lowest solubilization efficiency (113.14 ± 1.19 µg/mL). The superior performance of Solutol^®^ HS 15 can be attributed to its amphiphilic structure, which consists of PEG esters of 12-hydroxystearic acid [30]. This configuration provides a large and flexible hydrophobic region capable of accommodating lipophilic molecules such as REB. Tween^®^ 80 also demonstrated strong solubilizing ability, primarily due to its long C18 oleic acid chain, which forms a larger hydrophobic core than Tween^®^ 20 (C12) [31,32]. This structural difference is reflected in their solubility values, Tween^®^ 80 nearly doubled the solubility achieved by Tween^®^ 20 (133.24 ± 1.26 µg/mL) [33]. A similar pattern was observed with Pluronic^®^ surfactants. Pluronic^®^ F127 (212.85 ± 0.73 µg/mL) enhanced solubility to a greater extent than Pluronic^®^ F68, which can be explained by the higher proportion of hydrophobic POP blocks in F127. Larger POP segments contribute to a more hydrophobic micellar core, offering greater space for accommodating REB. This trend is consistent with previously reported findings for other hydrophobic drugs [34]. Labrasol^®^ produced moderate enhancement (170.91 ±0.55 µg/mL), which aligns with its ability to form mixed micellar or microemulsion-like aggregates. However, its solubilization potential was still lower than that of Solutol^®^ HS 15 and Tween^®^ 80. The solubility data indicate that micellar solubilization of REB depends strongly on the surfactant’s hydrophobic chain length, flexibility, and POP/PEG balance.

### 3.2. Stability During Dilution

The dilution-induced stability of REB-loaded micelles was evaluated to simulate the immediate exposure of ocular inserts to tear fluid during wear. Saturated drug-loaded micellar solutions were diluted 10- and 100-fold with STF, and percentage transmittance was measured as an indicator of micellar integrity and drug precipitation. At 10-fold dilution, all surfactant-based formulations exhibited high transmittance values (>97%), indicating that dilution did not induce significant turbidity or precipitation of REB. Minor reductions in transmittance were observed across formulations attributed to transient micellar rearrangement in the ionic tear-simulating medium rather than micelle destabilization. Pluronic^®^ F127 and Pluronic^®^ F68 formulations-maintained transmittance values of 97.47 ± 0.02% and 98.11 ± 0.01%, respectively, reflecting their low critical micelle concentration and stable core–shell structure. Tween^®^-based systems (Tween^®^ 20 and Tween^®^ 80), along with Solutol^®^ HS 15 and Labrasol^®^, also retained optical clarity, demonstrating adequate solubilization of REB under diluted conditions. Upon further dilution to 100-fold, all formulations exhibited near-complete transmittance (99.9–100%), suggesting that excess tear fluid did not promote drug precipitation and that residual surfactant molecules were sufficient to maintain REB in a solubilized state. The absence of transmittance loss or visible turbidity at both dilution levels confirms that REB-loaded micelles remain physically stable upon immediate exposure to tear fluid, minimizing the risk of dilution-induced micellar breakdown and burst drug release during ocular insert wear.

### 3.3. Size, Polydispersity Index, and Zeta Potential Analysis

**Figure 2:**
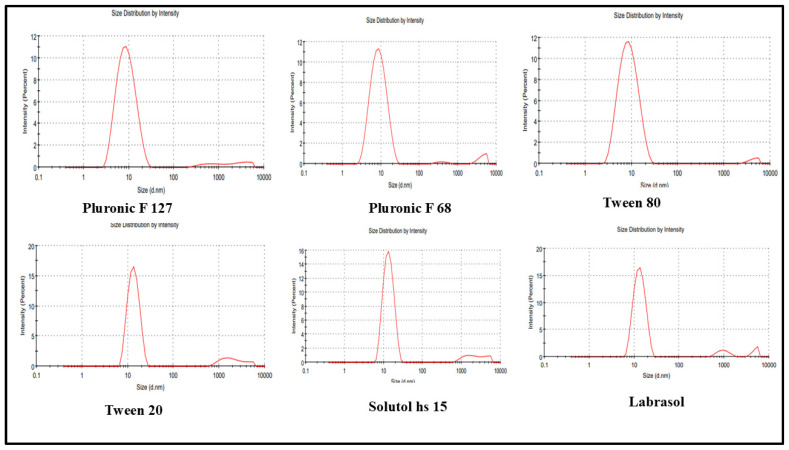
Mean particle size of micelles.

The particle size, polydispersity index (PDI), and zeta potential of the REB-loaded micellar systems were evaluated to understand the influence of surfactant type on micelle formation, colloidal stability, and suitability for ocular delivery. Dynamic light scattering analysis revealed that all formulations produced nanosized micelles, with mean particle sizes ranging from approximately 7.5 to 15.1 nm. The variation in mean particle size among the different surfactant-based micellar systems is illustrated in Figure 2. Pluronic-based micelles (Pluronic F127 and Pluronic F68) exhibited the smallest and most uniform particle sizes, both below 9 nm attributed to the efficient self-assembly of their amphiphilic block copolymer structure and the formation of a compact hydrophobic core capable of accommodating REB [34,35]. Tween 80–based micelles also demonstrated a small mean size (~7.5 nm), suggesting effective solubilization of REB within the micellar core. In contrast, Tween 20, Solutol HS 15, and Labrasol produced relatively larger micelles (~14–15 nm), likely due to differences in hydrophilic–lipophilic balance and molecular packing at the micellar interface. The PDI values for all formulations were below 0.35, indicating narrow size distributions and a high degree of homogeneity. Pluronic F68 and Tween 80 micelles showed the lowest PDI values, reflecting superior uniformity and minimal aggregation. Such low PDI values are particularly desirable for ocular applications, as they ensure consistent drug distribution and predictable release behavior from the ocular insert matrix. In addition, a slightly higher PDI observed for Tween 20 and Labrasol formulations may be associated with less compact micellar organization; however, the values remained within acceptable limits for nanoscale drug delivery systems. Zeta potential measurements indicated that all REB-loaded micelles possessed near-neutral to mildly negative surface charges, with values ranging from approximately −0.08 to −2.81 mV. This behavior is characteristic of non-ionic surfactant–based micellar systems, where steric stabilization rather than electrostatic repulsion plays a dominant role in maintaining colloidal stability. Despite the low absolute zeta potential values, no visible aggregation or precipitation was observed during measurements, suggesting that the micelles remained physically stable. From an ocular delivery perspective, a near-neutral surface charge is advantageous, as it minimizes the risk of ocular irritation and reduces nonspecific interactions with the negatively charged ocular surface [36,37]. The particle size, PDI, and zeta potential results confirm the successful formation of stable, nanosized REB-loaded micelles across all surfactant systems studied. Among them, Pluronic F127, Pluronic F68, and Tween 80 demonstrated the most favorable physicochemical characteristics in terms of small particle size and low PDI, supporting their suitability for incorporation into micellar ocular inserts. These attributes are expected to contribute to improved drug solubilization, uniform drug loading, and consistent performance of the ocular insert, which are critical for the enhanced management of dry eye disease.

### 3.4. Sterilization and Sterility Testing

All six ocular inserts subjected to moist heat sterilization at 121 °C under 15 psi pressure for 20 min remained physically intact, with no observable changes in shape, color, or surface characteristics following autoclaving in STF. Sterility testing conducted using the direct inoculation method demonstrated that none of the six inserts produced turbidity or visible microbial growth in either fluid thioglycollate medium or soybean–casein digest medium during the 14-day incubation period. Both media remained clear throughout the observation period, indicating the absence of viable anaerobic and aerobic microorganisms. The uninoculated control media also showed no signs of turbidity, confirming the adequacy of the culture media and incubation conditions. These results establish that the applied moist heat sterilization process was effective in achieving sterility across all six ocular inserts without compromising their physical integrity, supporting their suitability for subsequent evaluation and ocular application.

### 3.5. Physicochemical Evaluation of Ocular Inserts

#### 3.5.1. Appearance, Thickness, and Weight Uniformity

All fabricated ocular inserts were visually examined to assess their physical appearance prior to further evaluation. The inserts were transparent, smooth, and free from visible defects such as air bubbles, cracks, or particulate matter. The uniform surface morphology observed across all formulations indicates efficient photopolymerization and good compatibility between the polymeric matrix and the micelle-assisted drug loading process. The absence of surface irregularities is particularly important for ocular application, as it minimizes the risk of irritation and enhances patient comfort. Thickness uniformity is a critical parameter influencing mechanical stability, drug distribution, and release behavior of ocular inserts. The mean thickness of the inserts prepared using different surfactants ranged between 94.67 ± 0.58 and 98.33 ± 0.73 µm. Inserts loaded via surfactant systems exhibiting higher REB solubility showed marginally higher thickness values attributed to increased drug uptake within the polymeric network during the passive loading process. However, the observed variations were minimal, and the low standard deviation values confirm reproducible film formation and uniform crosslinking throughout the matrix. Weight uniformity followed a similar trend, with mean values ranging from 49.33 ± 1.53 to 50.67 ± 1.58 mg. All formulations complied with acceptable limits for mass variation, indicating consistent casting, curing, and post-loading handling of the inserts. Slight differences in weight among formulations can be linked to variations in drug incorporation efficiency arising from differences in micellar solubilization capacity. The consistent appearance, narrow thickness distribution, and uniform weight across all formulations confirm the reliability of the fabrication and micelle-assisted loading approach. These attributes are essential for ensuring reproducible drug content, predictable in vivo performance, and patient acceptability in ocular drug delivery applications.

#### 3.5.2. Surface pH

The surface pH of the REB-loaded ocular inserts was evaluated to assess their compatibility with the ocular surface environment. Following swelling in STF, all formulations exhibited surface pH values within a narrow range of 7.35 to 7.40, closely matching the physiological pH of tear fluid. This narrow variation indicates that the polymeric matrix and incorporated surfactants did not induce any significant pH alteration upon hydration. Inserts formulated using Pluronic F127, Pluronic F68, Tween 20, Tween 80, Solutol HS 15, and Labrasol showed comparable pH values, with minimal standard deviation, confirming the reproducibility of the measurement and uniform hydration behavior of the inserts. The slightly higher surface pH observed for Tween 80 and Solutol HS 15 based inserts may be attributed to their enhanced micellar solubilization capacity; however, the differences were negligible and remained well within the ocular tolerance range. Maintaining surface pH close to physiological conditions is critical to avoid ocular irritation, reflex tearing, or discomfort during application. The results demonstrate that the UV-curable polymer system, combined with micelle-assisted drug loading, preserves pH neutrality after swelling in tear fluid. This suggests that the prepared inserts are suitable for ocular administration and are unlikely to disrupt the natural tear film or corneal surface during residence in the conjunctival sac. The surface pH findings confirm the ocular safety of the developed inserts and support their further evaluation for sustained drug delivery applications.

#### 3.5.3. Moisture Absorption and Moisture Loss

The moisture absorption and moisture loss behavior of the ocular inserts was evaluated to understand their hygroscopic nature and stability under controlled humidity conditions. All formulations exhibited low moisture absorption, ranging from 1.82 ± 0.31% to 6.89 ± 0.87% when exposed to a relative humidity of approximately 67%. Inserts formulated with surfactants providing higher REB solubility demonstrated slightly increased moisture uptake attributed to enhanced hydrophilicity and greater interaction between the polymeric matrix and atmospheric moisture. Nevertheless, the overall moisture absorption remained within a narrow and acceptable range, indicating effective control of water uptake by the crosslinked polymer network. Moisture loss studies conducted under low-humidity conditions (RH ≈ 17%) revealed limited dehydration of the inserts, with moisture loss values ranging from 1.12 ± 0.02% to 3.73 ± 0.12%. A gradual increase in moisture loss was observed with increasing surfactant-mediated solubility, reflecting reversible moisture exchange rather than structural dehydration. Importantly, the magnitude of moisture loss was consistently lower than the corresponding moisture absorption, suggesting good water-retention capacity and resistance to brittleness during storage. The low and controlled moisture absorption and loss profiles confirm the physical stability of the ocular inserts under varying environmental conditions. Such balanced hygroscopic behavior is desirable for ocular drug delivery systems, as it ensures maintenance of dimensional integrity during storage while allowing appropriate hydration upon contact with tear fluid [38].

#### 3.5.4. Swelling Index

**Figure 3:**
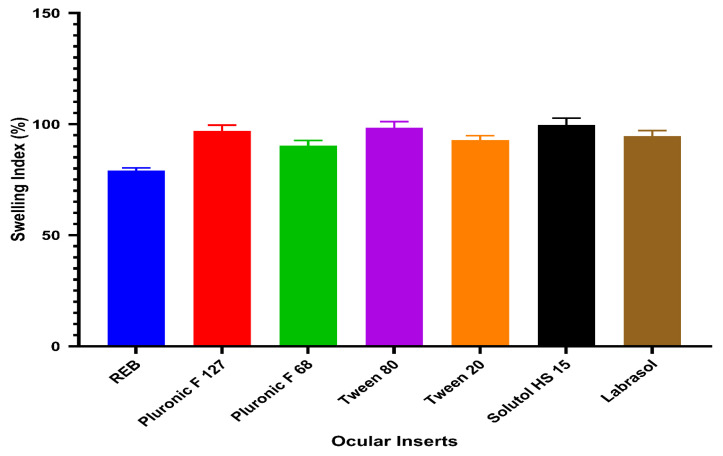
Swelling index of ocular inserts.

The swelling behavior of the ocular inserts was evaluated in STF to assess their hydration capacity under ocular conditions. As presented in Figure 3, all formulations exhibited pronounced swelling, with swelling index values ranging from 90.40 ± 2.21% to 99.60 ± 3.10%. This high degree of swelling reflects the hydrophilic nature of the polymeric matrix, which facilitates rapid water uptake upon contact with tear fluid. A gradual increase in swelling index was observed with the use of surfactants associated with higher REB solubilization capacity. Inserts prepared with Pluronic F68 showed the lowest swelling, whereas those containing Solutol HS 15 demonstrated the highest swelling extent. This trend suggests that micelle-assisted drug loading enhances water affinity within the polymer network, likely due to increased availability of hydrophilic domains and improved interaction with the aqueous medium. Despite the high swelling values, all inserts maintained their structural integrity throughout the study period, with no visible erosion or loss of shape. The relatively low standard deviation values further indicate uniform hydration behavior and reproducibility across samples. Such controlled swelling is desirable for ocular inserts, as it promotes intimate contact with the ocular surface, improves comfort, and may facilitate sustained drug release without compromising mechanical stability. The swelling index results confirm that the developed ocular inserts possess adequate hydration capacity and are well suited for ocular drug delivery applications, supporting their further evaluation in release and performance studies.

#### 3.5.5. Folding Endurance

The folding endurance of the ocular inserts was evaluated to assess their mechanical strength and flexibility, which is essential attributes for safe handling and ocular comfort during application. All formulations exhibited high folding endurance values, ranging from 302 ± 9 to 346 ± 14 folds. These results indicate that the inserts possess sufficient flexibility to withstand repeated mechanical stress without cracking or breaking. Inserts formulated with Pluronic F68 showed the lowest folding endurance, whereas those containing Solutol HS 15 demonstrated the highest resistance to repeated folding. A gradual increase in folding endurance was observed with formulations incorporating surfactants associated with higher swelling capacity and improved polymer–surfactant interactions. This behavior may be attributed to enhanced chain mobility and uniform distribution of micelle-associated drug within the polymeric matrix, which contributes to improved mechanical integrity. Despite the differences among formulations, all inserts exceeded the minimum folding endurance typically considered acceptable for ocular films, confirming their robustness and suitability for ocular administration. The relatively low standard deviation values suggest consistent mechanical performance across samples, reflecting uniform curing and crosslinking during photopolymerization. The folding endurance results confirm that the developed ocular inserts combine adequate flexibility with mechanical stability, supporting their further evaluation as reliable platforms for sustained ocular drug delivery.

#### 3.5.6. In Vitro Degradation Study

The in vitro degradation behavior of the ocular inserts was evaluated by monitoring changes in the pH of STF during incubation at 37 °C over a period of seven days. The pH of the incubation medium remained within a narrow physiological range throughout the study, decreasing slightly from an initial value of 7.40 ± 0.02 to 7.35 ± 0.04 by the end of the incubation period. The gradual and minimal reduction in pH suggests that the polymeric matrix undergoes negligible degradation under simulated ocular conditions. The absence of abrupt pH changes indicates that no significant acidic or basic degradation by-products were released into the medium during the study period. This behavior can be attributed to the stable crosslinked network formed by the UV-curable monomer system, which limits hydrolytic breakdown and maintains chemical integrity in aqueous environments. Maintaining pH values close to physiological tear fluid is essential for ocular safety, as deviations may lead to irritation or discomfort. The observed pH stability confirms that the fabricated inserts are unlikely to disturb the ocular surface environment during residence in the conjunctival sac. Furthermore, the low variability in pH readings across time points reflects consistent material behavior and reproducibility of the degradation assessment. The in vitro degradation results demonstrate that the developed ocular inserts exhibit good physicochemical stability in STF over the evaluated period. This stability supports their suitability for sustained ocular drug delivery and justifies further investigation of their long-term performance and in vivo compatibility.

#### 3.5.7. Uniformity of Drug Content

**Figure 4:**
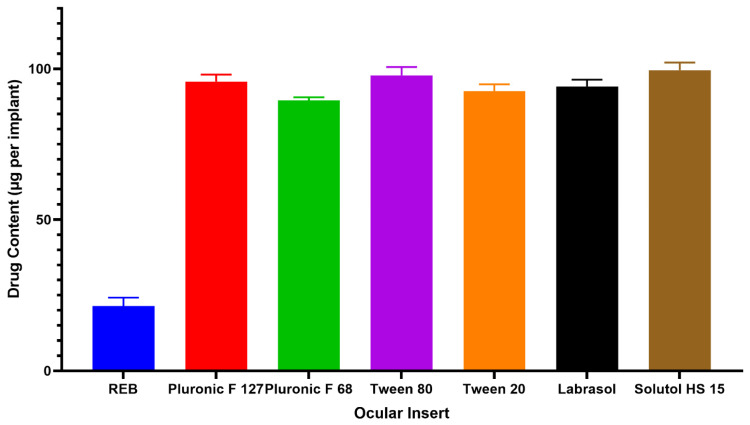
Uniformity of drug content.

The uniformity of drug content across ocular inserts was assessed to ensure consistent dosing and reproducible therapeutic performance (Table S6). As shown in Figure 4, plain REB-loaded inserts showed a markedly lower drug content (21.41 ± 2.28 µg per insert), which can be attributed to the poor aqueous solubility of REB and its limited diffusion into the polymeric matrix during post-fabrication loading. This observation underscores the intrinsic difficulty of achieving adequate and uniform drug loading when REB is incorporated in its free form. In contrast, micelle-assisted loading substantially enhanced drug incorporation, with drug content values ranging from 87.40 ± 3.25 to 99.19 ± 2.44 µg per insert depending on the surfactant system employed. The improved loading efficiency is primarily associated with micellar solubilization, which maintains REB in a molecularly dispersed state and promotes its diffusion and homogeneous distribution within the hydrophilic–siloxane polymer network. Among the evaluated formulations, inserts loaded using Solutol HS 15 and Tween 80 micelles exhibited the highest drug content, consistent with their superior solubilization capacity for REB. Furthermore, the relatively low standard deviation values observed across all micellar formulations indicate good batch uniformity and reproducibility of the loading process. Collectively, these findings confirm that micelle-assisted passive loading effectively overcomes the solubility limitations of REB, enabling uniform drug incorporation and supporting the suitability of the developed ocular inserts for sustained ocular drug delivery.

#### 3.5.8. Drug Leaching During Sterilization of REB-Loaded Ocular Inserts

**Figure 5:**
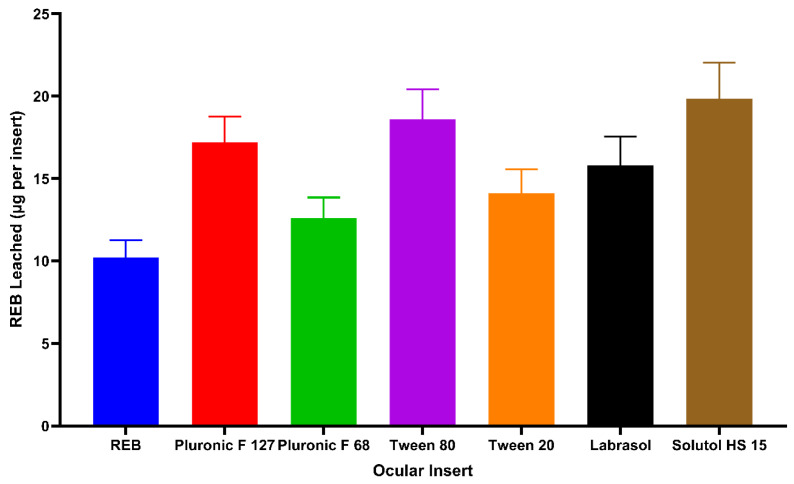
Drug leaching during sterilization.

The effect of autoclave sterilization on REB content in ocular inserts was evaluated to determine the extent of drug loss during moist heat treatment (Table S7). As shown in Figure 5, drug leaching from the inserts ranged from 10.20 ± 0.87 µg per implant for plain REB inserts to 19.83 ± 1.81 µg per implant for inserts loaded with Solutol HS 15 micelles. A clear trend was observed whereby formulations containing surfactants with higher drug solubilization capacity exhibited greater drug loss during sterilization. Inserts prepared with Solutol HS 15 and Tween 80, which provided the highest solubility for REB, showed the highest leaching values, while inserts with lower solubility surfactants or plain REB demonstrated comparatively lower drug loss. This indicates that the fraction of drug present in a solubilized or loosely bound state within the polymer matrix is more susceptible to migration during exposure to high temperature and pressure. Despite this leaching, all inserts retained the majority of their drug load, suggesting that the polymeric network effectively limits drug migration and maintains functional dosing after sterilization. The low standard deviation values across formulations indicate reproducible behavior and uniform distribution of drug within the inserts.

#### 3.5.9. In Vitro Release Studies

**Figure 6:**
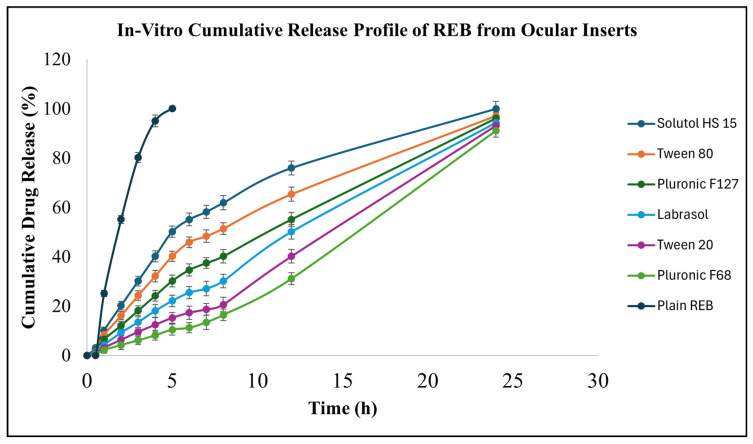
In vitro cumulative release profile of REB from ocular inserts.

The in vitro release profiles of REB from micelle-assisted and plain drug-loaded ocular inserts demonstrated a clear distinction in release behavior (Figure 6, Tables S8–S10). The release data are expressed as cumulative percentage drug release. However, all values were derived from experimentally measured drug release in micrograms (µg) and subsequently normalized with respect to the actual drug content remaining after sterilization. The total drug content of each formulation was initially determined by assay prior to sterilization. Following sterilization, a measurable loss of drug was observed. Therefore, the net remaining drug content in each formulation was re-evaluated and considered as the effective drug load for subsequent release studies. The post-sterilization drug content was found to be 11.20 µg for plain REB inserts, whereas micelle-assisted formulations retained significantly higher amounts, namely 78.53 µg (Pluronic^®^ F127), 74.87 µg (Pluronic^®^ F68), 79.20 µg (Tween 80), 78.50 µg (Tween 20), 78.30 µg (Labrasol), and 79.37 µg (Solutol^®^ HS 15). These values were taken as 100% for their respective formulations to calculate cumulative drug release, thereby ensuring accurate comparison by accounting for sterilization-induced drug loss. Plain REB inserts exhibited a rapid release pattern, with 25.70 ± 0.85% of the drug released within 1 h (corresponding to 2.88 ± 0.10 µg) and reaching nearly complete release (100.72 ± 0.96%, i.e., 11.28 ± 0.11 µg) by 5 h. This immediate burst release can be attributed to the poor aqueous solubility of REB combined with its low drug loading, which prevents saturation of the release medium and allows rapid dissolution and diffusion of the entire drug content. Therefore, the release behavior of plain REB inserts is dose-limited rather than solubility-limited, resulting in faster release compared to micelle-based formulations. In contrast, micelle-assisted ocular inserts exhibited a markedly sustained release profile over 24 h. At 0.5 h, drug release ranged from 0.40 ± 0.03 to 2.47 ± 0.05 µg (0.54 ± 0.04% to 3.11 ± 0.06%), indicating minimal initial diffusion. By 5 h, cumulative drug release ranged from 7.85 ± 0.45 to 39.92 ± 0.65 µg (10.48 ± 0.60% to 50.30 ± 0.82%), demonstrating effective delay of drug release. A gradual increase in release was observed during the intermediate period, and near-complete release was achieved at 24 h, with values ranging from 69.07 ± 1.23 to 79.77 ± 0.87 µg (92.25 ± 1.64% to 100.50 ± 1.10%).

The sustained release behavior of micellar formulations can be attributed to drug encapsulation within micelles and controlled diffusion from the micelle–polymer network. Drug release occurs through a combination of diffusion of free drug and gradual dissociation of drug from micellar structures, which slows down the release rate. Similar mechanisms have been reported in micelle-laden hydrogel systems, where an initial limited burst release is followed by sustained drug release due to drug–micelle and drug–polymer interactions, as reported by Hu et al. [39]. Furthermore, the presence of micelles introduces an additional diffusion barrier, wherein the drug must first partition from the micellar core into the surrounding polymer matrix and subsequently into the release medium, thereby prolonging the release duration. This diffusion-controlled mechanism has been well documented by Yang et al. [40].

The differences in release behavior among micellar formulations can be attributed to variations in solubilization capacity and micellar characteristics of the surfactants. Surfactants such as Solutol^®^ HS 15 and Tween 80 exhibited relatively higher drug release, likely due to enhanced solubilization and dynamic partitioning of the drug into the aqueous phase. In contrast, Pluronic-based systems and other surfactants demonstrated comparatively slower release, which may be attributed to greater micellar stability and stronger drug retention within the hydrophobic core. These observations are consistent with previous findings reported by Furqan A. Maulvi et al. [41]. The low standard deviation values confirm good reproducibility of the release profiles. Overall, micelle-assisted inserts enabled sustained and controlled drug release, whereas plain REB inserts exhibited rapid, dose-dependent release due to low drug loading and absence of solubilization mechanisms.

#### 3.5.10. Drug Release Kinetics and Mechanistic Modeling

The release kinetics of REB from micelle-assisted and plain ocular inserts were systematically evaluated using multiple mathematical models to elucidate the dominant drug-release mechanism (Table 1). The fabricated inserts, prepared via UV-induced photopolymerization and post-fabrication passive loading, provided a stable polymeric matrix suitable for controlled diffusion-based drug delivery. The zero-order kinetic model exhibited comparatively low regression coefficients for most formulations (R^2^ = 0.665–0.996), with particularly poor fitting for the plain REB insert (R^2^ = −0.476), indicating that drug release did not occur at a constant rate. This suggests that simple matrix-controlled zero-order kinetics were insufficient to describe the release behavior, especially in the absence of solubilizing carriers. In contrast, the first-order model showed high correlation coefficients for all micelle-assisted formulations (R^2^ = 0.798 to 0.994) and the plain REB insert (R^2^ = 0.951), indicating a concentration-dependent release process. This behavior is consistent with diffusion-controlled release from hydrated polymer matrices, where the release rate decreases as the drug concentration within the insert diminishes. The Higuchi model demonstrated good linearity for micelle-assisted inserts containing Solutol HS 15 and Tween 80 (R^2^ > 0.95), supporting Fickian diffusion as a major contributor during the early and intermediate phases of drug release. However, a progressive decline in Higuchi correlation was observed for Pluronic F68 and plain REB inserts, suggesting deviation from pure diffusion due to matrix relaxation effects or rapid drug depletion in the absence of micellar stabilization. The Hixson–Crowell model showed excellent correlation for micelle-assisted formulations (R^2^ = 0.835–0.992), indicating that changes in surface area and polymer matrix geometry contributed to the release process. The lower correlation observed for the plain REB insert (R^2^ = 0.738) suggests uncontrolled matrix erosion or rapid dissolution-driven release, consistent with its burst-release profile. The Korsmeyer–Peppas model provided the most mechanistic insight into the release behavior. The diffusion exponent (*n*) values for micelle-assisted inserts ranged from 0.558 to 1.486, indicating a transition from anomalous transport (Solutol HS 15, Tween 80, Pluronic F127) to Case-II and Super Case-II transport (Labrasol, Tween 20, Pluronic F68). This behavior reflects the combined influence of drug diffusion, polymer chain relaxation, and swelling of the UV-crosslinked hydrogel matrix. In contrast, the plain REB insert exhibited an n value of 0.303, characteristic of Fickian diffusion, confirming rapid drug diffusion without matrix-controlled modulation. Kinetic modeling clearly demonstrates that micelle-assisted loading significantly alters the release mechanism of REB by promoting controlled diffusion and polymer-relaxation-mediated transport, whereas plain REB inserts predominantly exhibit rapid diffusion-driven release. The superior fitting of first-order, Hixson–Crowell, and Korsmeyer–Peppas models for micellar formulations confirms the role of micellar solubilization and polymer–drug interactions in achieving sustained ocular drug delivery. These findings strongly support the suitability of micelle-assisted ocular inserts for prolonged therapy in dry eye disease, with the potential to reduce dosing frequency and improve patient compliance.

#### 3.5.11. Ex Vivo Transcorneal Permeation and Corneal Deposition Studies

**Figure 7:**
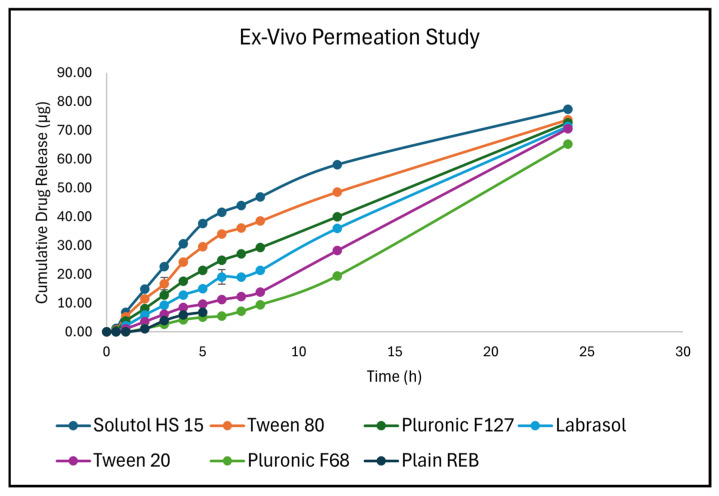
Ex Vivo Drug Permeation Profile.

The transcorneal permeation of REB from the developed micellar ocular inserts was evaluated using excised goat corneas mounted on Franz diffusion cells. All formulations exhibited a time-dependent increase in cumulative drug permeation over 24 h (Figure 7), confirming sustained drug release behavior from the inserts. The micellar systems significantly enhanced REB permeation compared to the plain drug formulation, indicating the beneficial role of surfactants in improving solubilization and corneal transport. Among the tested formulations, Solutol HS 15 demonstrated the highest cumulative permeation, reaching 77.30 ± 0.34 µg at 24 h, followed by Tween 80 (73.69 ± 0.60 µg) and Pluronic F127 (72.68 ± 1.12 µg). Moderate permeation was observed with Labrasol (71.34 ± 0.93 µg) and Tween 20 (70.50 ± 0.73 µg), while Pluronic F68 showed the lowest permeation (65.20 ± 0.46 µg) among the micellar systems. In contrast, the plain REB formulation exhibited comparatively lower and incomplete permeation over the study duration, indicating limited corneal transport without micellar assistance. The steady-state flux (Jss), calculated from the linear portion of the permeation profile (typically 2–6 h), clearly differentiated the performance of the formulations. Solutol HS 15 exhibited the highest flux (8.52 µg/cm^2^·h), followed by Tween 80 (7.17 µg/cm^2^·h) and Pluronic F127 (5.32 µg/cm^2^·h). Lower flux values were observed for Labrasol (4.19 µg/cm^2^·h), Tween 20 (2.42 µg/cm^2^·h), and Pluronic F68 (1.38 µg/cm^2^·h). The plain REB formulation showed a flux of 1.82 µg/cm^2^·h, which was lower than most micellar systems, confirming the role of surfactants in enhancing drug permeation. Similarly, the permeation rate (dQ/dt) followed the same trend, with Solutol HS 15 (6.69 µg/cm^2^·h) showing the highest value, followed by Tween 80 (5.63 µg/cm^2^·h) and Pluronic F127 (4.14 µg/cm^2^·h). The lowest permeation rate was observed with Pluronic F68 (1.08 µg/cm^2^·h), indicating comparatively weaker permeation enhancement. The enhanced permeation observed with micellar formulations can be attributed to improved drug solubilization, reduction in diffusional resistance, and possible interactions of surfactants with the corneal epithelium, leading to increased membrane permeability. Among all formulations, Solutol HS 15 and Tween 80 emerged as the most effective permeation enhancers, providing both higher cumulative permeation and flux. The ex vivo permeation results demonstrate that micellar ocular inserts significantly improve transcorneal delivery of REB compared to the plain formulation, with performance strongly dependent on the type of surfactant used. These findings highlight the potential of surfactant-based micellar systems for sustained and enhanced ocular drug delivery. A comparison between the in vitro drug release and ex vivo permeation profiles revealed a consistent correlation between the two studies. Formulations exhibiting higher in vitro release also demonstrated enhanced transcorneal permeation, indicating that improved drug availability from the formulation contributes directly to permeation efficiency. However, the extent of drug permeation was relatively lower than in vitro release, which can be attributed to the barrier properties of the corneal membrane. The sustained release pattern observed in vitro was well reflected in the ex vivo study, confirming controlled drug delivery behavior. The findings suggest that in vitro release data can serve as a reliable predictor of ex vivo permeation performance.

### 3.6. Cytocompatibility

**Figure 8:**
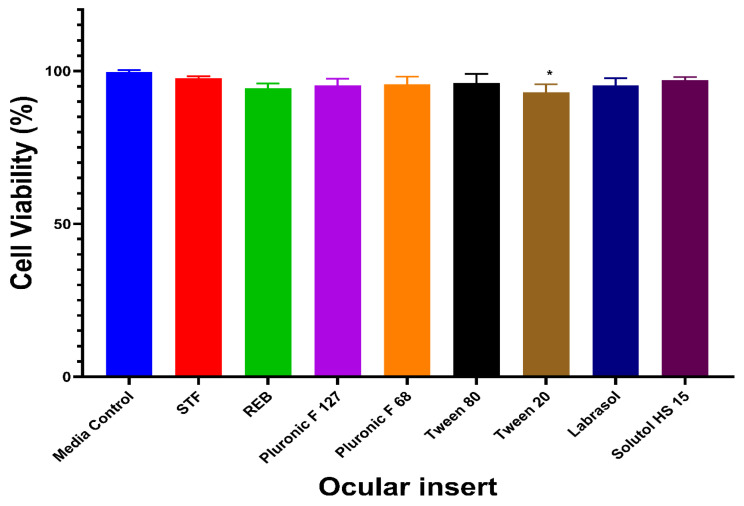
Cytocompatibility assessment of micelle-laden ocular inserts by MTT assay (* *p* < 0.05 compared to the media control group).

The cytocompatibility of the developed micelle-laden ocular inserts was evaluated using the MTT assay on SIRC cells, and cell viability was expressed as a percentage relative to the media control (Figure 8, Tables S11–S13). The media control exhibited a cell viability of 99.67 ± 0.58%, confirming optimal cell growth conditions. Cells treated with STF also showed high viability (97.67 ± 0.58%), validating its suitability as the extraction medium. Among the tested formulations, the REB-loaded ocular insert demonstrated a cell viability of 94.33 ± 1.53%, indicating good cytocompatibility with no observable cytotoxic effects. Ocular inserts formulated using Pluronic F127-based and Pluronic F68-based micelles exhibited cell viabilities of 95.33 ± 2.08% and 95.67 ± 2.52%, respectively. Similarly, Tween 80- and Tween 20-based micelle-laden ocular inserts showed high cell viability values of 96.00 ± 3.00% and 92.00 ± 2.65%, respectively. Ocular inserts incorporating Labrasol-based and Solutol HS 15-based micelles demonstrated excellent cytocompatibility, with cell viability values of 95.33 ± 2.31% and 97.00 ± 1.00%, respectively. Overall, all micelle-laden ocular inserts-maintained cell viability above 90%, well exceeding the accepted cytocompatibility threshold. These results confirm that the developed micelle-based ocular inserts are non-cytotoxic to SIRC cells and suitable for ocular drug delivery applications.

### 3.7. Histopathological Evaluation

**Figure 9:**
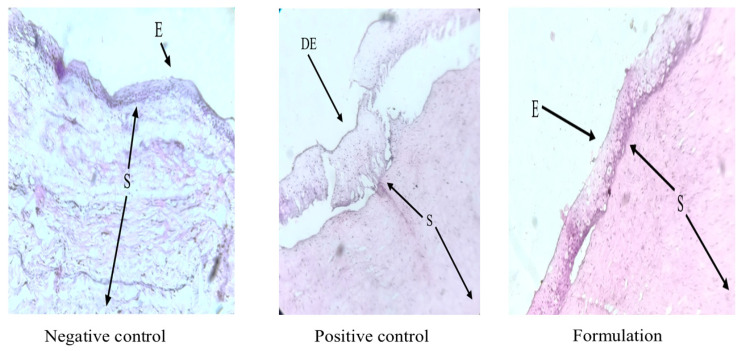
Histopathological evaluation of goat corneal tissue after 24 h exposure to different treatments (E = Epithelium; DE = Damaged Epithelium; S = Stroma).

Histopathological examination of goat corneal tissues stained with hematoxylin and eosin (H&E) after 24 h of exposure to different treatments is presented in Figure 9. The negative control group (tear fluid-treated) exhibited normal corneal architecture, with well-organized and intact epithelial and stromal layers, indicating the preservation of physiological morphology. In contrast, the positive control group (absolute alcohol-treated) showed evident structural damage, characterized by disruption and degeneration of the epithelial layer along with alterations in the underlying stromal region. Notably, the formulation-treated group demonstrated well-preserved corneal morphology, with intact epithelial and stromal layers comparable to those observed in the negative control group. No significant signs of epithelial erosion, cellular disorganization, or stromal damage were observed in this group. The absence of observable structural alterations in the formulation-treated corneas suggests that the developed formulation is non-irritant and exhibits good ocular compatibility. The maintenance of normal corneal architecture further indicates that the formulation does not induce cytotoxic effects or morphological damage under the tested conditions, supporting its suitability for ophthalmic application.

## 4. Conclusions

This work establishes micelle-assisted ocular inserts as a viable and advanced platform for the sustained delivery of poorly water-soluble drugs such as REB. Micellar incorporation fundamentally transformed the delivery behavior of REB by enabling stable solubilization, controlled diffusion, and prolonged ocular residence within a UV-crosslinked polymeric matrix. The developed inserts demonstrated consistent physicochemical stability, predictable swelling–release behavior, and mechanical robustness suitable for ocular application. Importantly, micelle-mediated modulation of drug transport resulted in sustained transcorneal permeation and measurable corneal deposition, while maintaining excellent cytocompatibility with corneal epithelial cells. Among the evaluated systems, Solutol HS 15 and Tween 80 provided the most favorable balance between solubilization efficiency, release control, and ocular compatibility. Overall, micelle-assisted ocular inserts represent a promising, patient-friendly alternative to conventional eye drops, with strong potential for improving therapeutic outcomes in chronic dry eye disease.

## Figures and Tables

**Table 1:** Drug release kinetics and mechanistic modeling of REB-loaded ocular inserts.

Model	Parameter	Solutol HS 15	Tween 80	Pluronic F127	Labrasol	Tween 20	Pluronic F68	Plain REB	Mean	SD	RSD (%)
Zero-order (Qt vs. t)	K_0_	5.517	4.961	4.431	4.000	3.559	3.212	7.530	4.744	1.461	30.80
	R^2^	0.665	0.816	0.952	0.996	0.973	0.920	−0.476	—	—	—
First-order	R^2^	0.994	0.991	0.972	0.933	0.862	0.798	0.951	—	—	—
Higuchi	kH	20.728	18.168	15.619	13.559	11.509	9.972	31.523	17.297	7.290	42.15
	R^2^	0.958	0.951	0.894	0.802	0.680	0.584	0.606	—	—	—
Hixson–Crowell	kHC	0.036	0.028	0.022	0.018	0.014	0.012	0.069	—	—	—
	R^2^	0.991	0.992	0.989	0.963	0.899	0.835	0.738	—	—	—
Korsmeyer–Peppas	kKP	18.142	13.175	8.022	4.485	1.929	0.807	48.997	13.651	16.772	122.87
	n	0.558	0.639	0.782	0.959	1.219	1.486	0.303	0.849	0.405	47.74
	R^2^	0.957	0.982	0.997	0.997	0.995	0.997	0.650	—	—	—

## Data Availability

The data presented in this study are contained within the article. Further inquiries can be directed to the corresponding author.

## Supplementary Materials

The following supporting information can be downloaded at: https://www.mdpi.com/article/10.3390/pharmaceutics18050578/s1. Figure S1: Calibration curve of rebamipide in methanol; Figure S2: Calibration curve of rebamipide in simulated tear fluid (STF); Figure S3: UV–visible absorption spectrum of rebamipide in methanol; Figure S4: UV–visible absorption spectrum of rebamipide in STF; Figure S5: Overlay spectra of rebamipide micellar solution and plain Solutol HS15 solution; Figure S6: Overlay spectra of rebamipide micellar solution and plain Tween 80 solution; Figure S7: Overlay spectra of rebamipide micellar solution and plain Tween 20 solution; Figure S8: Overlay spectra of rebamipide micellar solution and Pluronic F127 solution; Figure S9: Overlay spectra of rebamipide micellar solution and Pluronic F68 solution; Figure S10: Overlay spectra of rebamipide micellar solution and Labrasol solution; Figure S11: Overlay spectra of rebamipide solution and ocular insert extract after 24 h. Table S1: Linear regression analysis of calibration curves of rebamipide in methanol and STF; Table S2: Precision study data (intra-day and inter-day); Table S3: Accuracy (% recovery) study; Table S4: Limit of detection (LOD) and limit of quantification (LOQ); Table S5: Saturation solubility data; Table S6: Assay of rebamipide-loaded ocular inserts; Table S7: Drug leaching during sterilization; Tables S8–S10: In vitro drug release data; Tables S11–S13: Ex vivo permeation data.

## References

[1] Messmer E.M. (2015). The Pathophysiology, Diagnosis, and Treatment of Dry Eye Disease. Dtsch. Arztebl. Int..

[2] Mohamed H.B., Abd El-Hamid B.N., Fathalla D., Fouad E.A. (2022). Current Trends in Pharmaceutical Treatment of Dry Eye Disease: A Review. Eur. J. Pharm. Sci..

[3] Perez V.L., Stern M.E., Pflugfelder S.C. (2020). Inflammatory Basis for Dry Eye Disease Flares. Exp. Eye Res..

[4] Gayton J. (2009). Etiology, Prevalence, and Treatment of Dry Eye Disease. Clin. Ophthalmol..

[5] Nagai N., Otake H. (2022). Novel Drug Delivery Systems for the Management of Dry Eye. Adv. Drug Deliv. Rev..

[6] Zhang X., M V.J., Qu Y., He X., Ou S., Bu J., Jia C., Wang J., Wu H., Liu Z. (2017). Dry Eye Management: Targeting the Ocular Surface Microenvironment. Int. J. Mol. Sci..

[7] Priyavarshini R., Amit B. (2020). Patil Recent Development in the Artificial Treatment for Dry Eye Disease. Int. J. Res. Pharm. Sci..

[8] (2015). Improving Diagnosis and Outcomes of Sjögren’s Disease through Targeting Dry Eye Patients: A Continuing Medical Education Enduring Material. Ocul. Surf..

[9] Phadatare S.P., Momin M., Nighojkar P., Askarkar S., Singh K.K. (2015). A Comprehensive Review on Dry Eye Disease: Diagnosis, Medical Management, Recent Developments, and Future Challenges. Adv. Pharm..

[10] Aragona P., Barabino S., Di Zazzo A., Giannaccare G., Villani E., Aiello F., Antoniazzi E., Bonini S., Cantera E., Carlini G. (2025). Dry Eye Disease: From Causes to Patient Care and Clinical Collaboration—A Narrative Review. Ophthalmol. Ther..

[11] Kwon J., Moghtader A., Kang C., Bibak Bejandi Z., Shahjahan S., Alzein A., Djalilian A.R. (2025). Overview of Dry Eye Disease for Primary Care Physicians. Medicina.

[12] Agarwal P., Craig J.P., Rupenthal I.D. (2021). Formulation Considerations for the Management of Dry Eye Disease. Pharmaceutics.

[13] Kawahara A. (2023). Treatment of Dry Eye Disease (DED) in Asia: Strategies for Short Tear Film Breakup Time-Type DED. Pharmaceutics.

[14] Kashima T., Akiyama H., Kishi S., Itakura H. (2014). Rebamipide Ophthalmic Suspension for the Treatment of Dry Eye Syndrome: A Critical Appraisal. Clin. Ophthalmol..

[15] Jiang E., Jin H., Liu J., Kim H.J., Yoon H.S., Choi J.S., Moon J., Choi H.-I., Yoon H.-J., Yoon K.C. (2025). Comparison of the Therapeutic Effects of Rebamipide and Diquafosol on Apoptotic Damage of the Ocular Surface in Dry Eyes. Antioxidants.

[16] Qiao H., Xu Z., Sun M., Fu S., Zhao F., Wang D., He Z., Zhai Y., Sun J. (2022). Rebamipide Liposome as an Effective Ocular Delivery System for the Management of Dry Eye Disease. J. Drug Deliv. Sci. Technol..

[17] Eom Y., Chung S.H., Chung T.-Y., Kim J.Y., Choi C.Y., Yoon K.C., Ko B.Y., Kim H.K., Kim M.K., Lee H.K. (2023). Efficacy and Safety of 1% and 2% Rebamipide Clear Solution in Dry Eye Disease: A Multicenter Randomized Trial. BMC Ophthalmol..

[18] Simsek C., Kojima T., Nakamura S., Dogru M., Tsubota K. (2019). The Effects of Rebamipide 2% Ophthalmic Solution Application on Murine Subbasal Corneal Nerves After Environmental Dry Eye Stress. Int. J. Mol. Sci..

[19] Jang D.-J., Lee J.H., Kim D.H., Kim J.-W., Koo T.-S., Cho K.H. (2023). The Development of Super-Saturated Rebamipide Eye Drops for Enhanced Solubility, Stability, Patient Compliance, and Bioavailability. Pharmaceutics.

[20] Nagai N., Ishii M., Seiriki R., Ogata F., Otake H., Nakazawa Y., Okamoto N., Kanai K., Kawasaki N. (2020). Novel Sustained-Release Drug Delivery System for Dry Eye Therapy by Rebamipide Nanoparticles. Pharmaceutics.

[21] Otake H., Kobayashi K., Kadowaki R., Kosaka T., Itahashi M., Tsubaki M., Matsuda M., Iwakiri N., Harata E., Nagai N. (2024). Copolymerized Polymers Based on Cyclodextrins and Cationic Groups Enhance Therapeutic Effect of Rebamipide in the N-Acetylcysteine-Treated Dry Eye Model. Drug Des. Dev. Ther..

[22] Ghezzi M., Ferraboschi I., Delledonne A., Pescina S., Padula C., Santi P., Sissa C., Terenziani F., Nicoli S. (2022). Cyclosporine-Loaded Micelles for Ocular Delivery: Investigating the Penetration Mechanisms. J. Control. Release.

[23] Mariz M., Murta J., Gil M.H., Ferreira P. (2022). An Ocular Insert with Zero-Order Extended Delivery: Release Kinetics and Mathematical Models. Eur. J. Pharm. Biopharm..

[24] Di Prima G., Licciardi M., Carfì Pavia F., Lo Monte A.I., Cavallaro G., Giammona G. (2019). Microfibrillar Polymeric Ocular Inserts for Triamcinolone Acetonide Delivery. Int. J. Pharm..

[25] Alambiaga-Caravaca A.M., Domenech-Monsell I.M., Sebastián-Morelló M., Calatayud-Pascual M.A., Merino V., Rodilla V., López-Castellano A. (2021). Development, Characterization, and Ex Vivo Evaluation of an Insert for the Ocular Administration of Progesterone. Int. J. Pharm..

[26] Karnik I., Youssef A.A.A., Joshi P., Munnangi S.R., Narala S., Varner C., Vemula S.K., Majumdar S., Repka M. (2023). Formulation Development and Characterization of Dual Drug Loaded Hot-Melt Extruded Inserts for Better Ocular Therapeutic Outcomes: Sulfacetamide/Prednisolone. J. Drug Deliv. Sci. Technol..

[27] Raj W., Jerczynski K., Rahimi M., Pavlova E., Šlouf M., Przekora A., Pietrasik J. (2022). Stimuli-Responsive Vitamin E-Based Micelles: Effective Drug Carriers with a Controlled Anticancer Drug Release. Polymer.

[28] Dyagala S., Paul M., Das S., Halder S., Biswas S., Saha S.K. (2025). Targeted Phototriggered and PH-Responsive Micellar Cancer Drug Delivery System with Real-Time Monitoring through the NSET Mechanism. ACS Appl. Bio Mater..

[29] Greenspan L. (1977). Humidity Fixed Points of Binary Saturated Aqueous Solutions. J. Res. Natl. Bur. Stand. A Phys. Chem..

[30] Younes N.F., Abdel-Halim S.A., Elassasy A.I. (2018). Solutol HS15 Based Binary Mixed Micelles with Penetration Enhancers for Augmented Corneal Delivery of Sertaconazole Nitrate: Optimization, in Vitro, Ex Vivo and in Vivo Characterization. Drug Deliv..

[31] Liang Z., Qiu X., Li J., Pan H., Liu H. (2024). An Investigation on the Hydrophobic Binding Stability of Azithromycin to the Hydrocarbon Chains of Tween: Based on Micellization Thermodynamics. J. Mol. Liq..

[32] Erawati T., Isadiartuti D., Anggalih B.D. (2023). The Effect of Polysorbate 20 and Polysorbate 80 on the Solubility of Quercetin. J. Public Health Afr..

[33] Weber J., Pedri L., Peters L.P., Quoika P.K., Dinu D.F., Liedl K.R., Tautermann C.S., Diederichs T., Garidel P. (2025). Micellar Solvent Accessibility of Esterified Polyoxyethylene Chains as Crucial Element of Polysorbate Oxidation: A Density Functional Theory, Molecular Dynamics Simulation and Liquid Chromatography/Mass Spectrometry Investigation. Mol. Pharm..

[34] Khaliq N.U., Lee J., Kim S., Sung D., Kim H. (2023). Pluronic F-68 and F-127 Based Nanomedicines for Advancing Combination Cancer Therapy. Pharmaceutics.

[35] Popovici C., Popa M., Sunel V., Atanase L.I., Ichim D.L. (2022). Drug Delivery Systems Based on Pluronic Micelles with Antimicrobial Activity. Polymers.

[36] Jiao J. (2008). Polyoxyethylated Nonionic Surfactants and Their Applications in Topical Ocular Drug Delivery. Adv. Drug Deliv. Rev..

[37] Wei Y., Mao Y., Zhu R., Xu Y., Qi Q., Wen X., Zhao J., Zhang J., Guan J., Zhang X. (2025). Intraocular Fate of Surface Charge-Dependent Nanomicelles via Topical Administration: Posterior Delivery and Transport Pathway. J. Control. Release.

[38] Kumari A., Sharma P., Garg V., Garg G. (2010). Ocular Inserts—Advancement in Therapy of Eye Diseases. J. Adv. Pharm. Technol. Res..

[39] Hu X., Tan H., Chen P., Wang X., Pang J. (2016). Polymer Micelles Laden Hydrogel Contact Lenses for Ophthalmic Drug Delivery. J. Nanosci. Nanotechnol..

[40] Yang H., Zhang F., Fan Y., Zhang J., Fang T., Xing D., Zhen Y., Nie Z., Liu Y., Wang D. (2024). Co-Delivery of Brinzolamide and Timolol from Micelles-Laden Contact Lenses: In Vitro and In Vivo Evaluation. Pharm. Res..

[41] Maulvi F.A., Parmar M.B., Shetty K.H., Patel A.R., Desai B.V., Vyas B.A., Desai D.T., Kalaiselvan P., Masoudi S., Shah D.O. (2024). Role of Micelle Dynamics in Enhancing Cyclosporine Uptake in Hyaluronic Acid-Contact Lenses for Improved Critical Lens Properties in Dry Eye Management. Colloids Surf. A Physicochem. Eng. Asp..

