# Supplementary material for: Micelle-Based Ocular Inserts for Sustained Delivery and Improved Corneal Permeation of Rebamipide in Dry Eye Disease
Source: Pharmaceutics. 2026 May 7;18(5):578. doi: 10.3390/pharmaceutics18050578 (PMC13210860; doi:10.3390/pharmaceutics18050578)
Supplement: Supplementary file 1 [file pharmaceutics-18-00578-s001.zip › pharmaceutics-4251332-supplementary.pdf]

Supplementary Materials

Table S1. Linear regression analysis of calibration curves of REB in Methanol and STF.

Concentration (µg/ml)	Absorbance ± SD (Methanol)	Absorbance ± SD (STF)
1	0.109 ± 0.0015	0.105 ± 0.0015
2	0.235 ± 0.0015	0.208 ± 0.0029
4	0.474 ± 0.0040	0.415 ± 0.0042
6	0.730 ± 0.0075	0.628 ± 0.0036
8	0.969 ± 0.0115	0.842 ± 0.0031
R^2	0.9999	0.9999
Intercept	0.123	0.1053

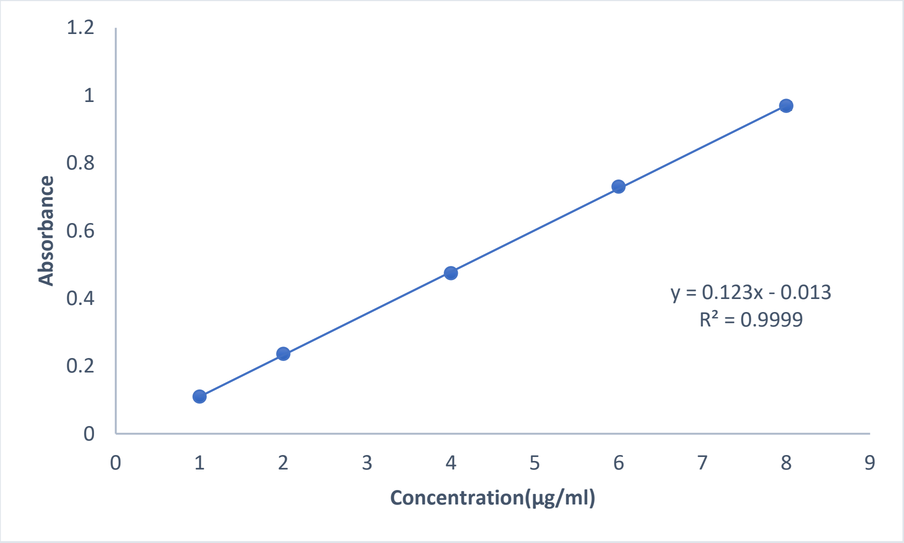

Figure S1. Calibration curve of Rebamipide in methanol.

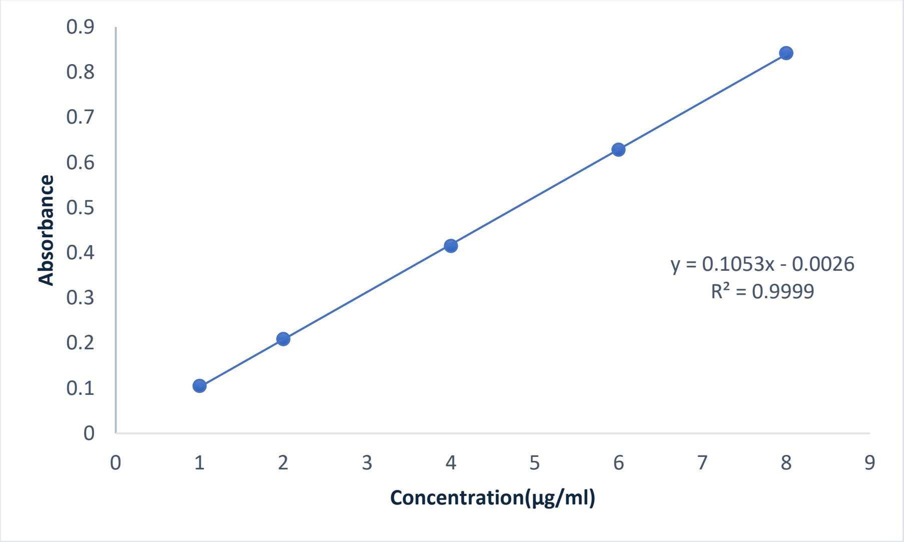

Figure S2. Calibration curve of Rebamipide in STF.

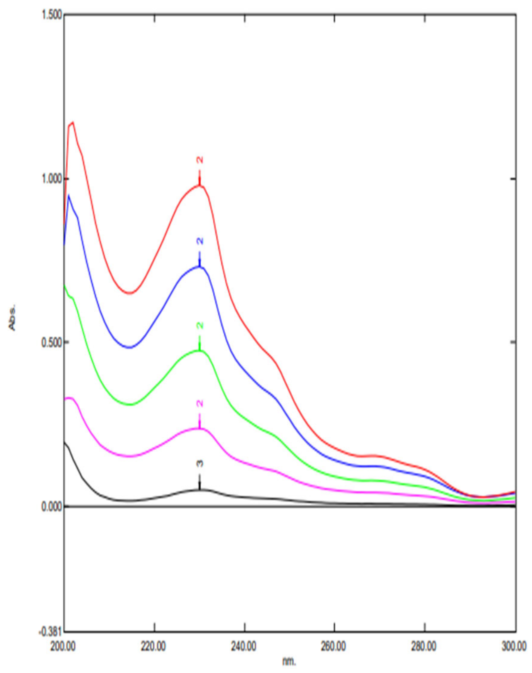

Figure S3. UV–visible absorption spectrum of rebamipide in methanol.

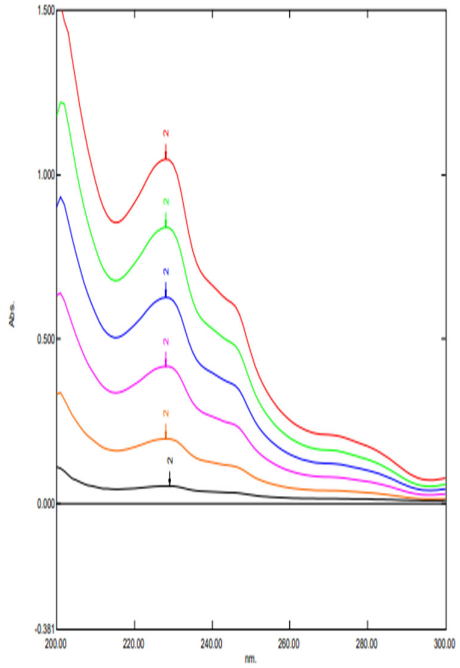

Figure S4. UV–visible absorption spectrum of rebamipide in STF.

Table S2. Data of precision study for REB.

Medium	Study Type	Conc. Taken (µg/mL)	Abs1	Abs2	Abs3	Mean Conc. ± SD (µg/mL)	% Recovery	%RSD
Methanol	Intraday	3	0.351	0.364	0.359	3.02 ± 0.05	100.54	0.05
		5	0.604	0.611	0.615	5.07 ± 0.05	101.30	0.04
		7	0.841	0.852	0.853	7.01 ± 0.05	100.08	0.05
	Interday	3	0.358	0.348	0.373	3.03 ± 0.10	100.99	0.10
		5	0.598	0.611	0.618	5.06 ± 0.08	101.14	0.08
		7	0.843	0.849	0.859	7.02 ± 0.07	100.27	0.07
STF	Intraday	3	0.318	0.323	0.310	3.04 ± 0.06	101.17	0.06
		5	0.520	0.537	0.524	5.03 ± 0.08	100.59	0.08
		7	0.731	0.738	0.749	7.05 ± 0.09	100.66	0.09
	Interday	3	0.319	0.310	0.324	3.04 ± 0.07	101.38	0.07
		5	0.523	0.524	0.539	5.05 ± 0.09	100.91	0.08
		7	0.728	0.739	0.751	7.05 ± 0.11	100.66	0.11

Table S3. Accuracy (% recovery) study of REB at three spiking levels.

Medium	Sample (µg/mL)	Spiked (µg/mL)	Expected (µg/mL)	Y1	Y2	Y3	Found (µg/mL ± SD)	% Recovery	% RSD
STF	4	3.2	7.2	0.755	0.759	0.748	7.19 ± 0.05	99.79	0.05
STF	4	4.0	8.0	0.841	0.839	0.848	8.03 ± 0.04	100.34	0.04
STF	4	4.4	8.8	0.923	0.915	0.918	8.75 ± 0.04	99.42	0.04
Methanol	4	3.2	7.2	0.873	0.879	0.869	7.21 ± 0.04	100.12	0.04
Methanol	4	4.0	8.0	0.978	0.971	0.964	8.00 ± 0.06	100.00	0.06
Methanol	4	4.4	8.8	1.071	1.070	1.061	8.78 ± 0.04	99.81	0.04

Specificity:

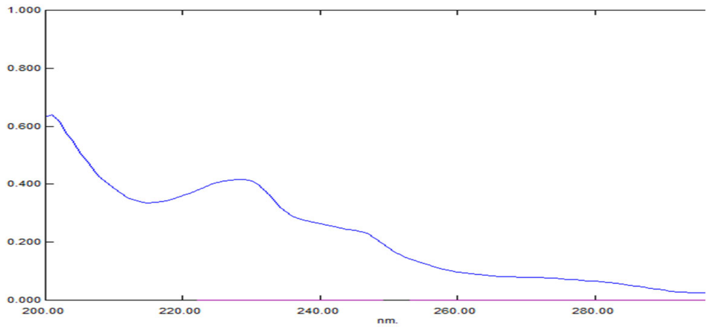

Figure S5. Overlay spectra of Rebamipide micellar solution and plain Solutol HS15 solution.

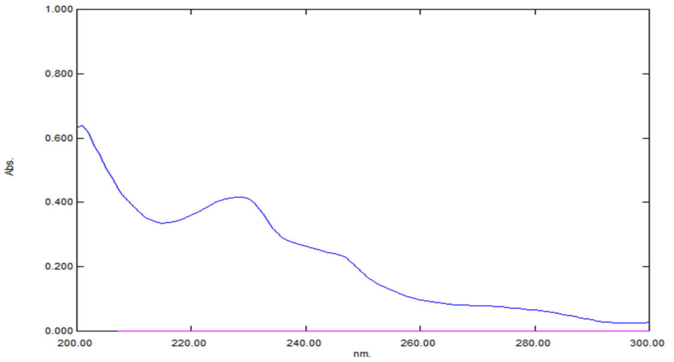

Figure S6. Overlay spectra of Rebamipide micellar solution and plain Tween 80 solution.

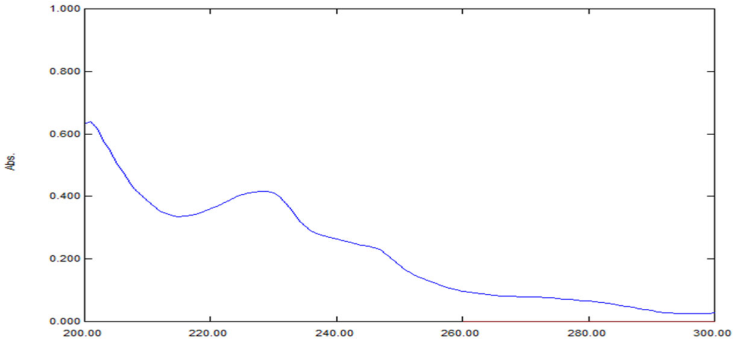

Figure S7. Overlay spectra of Rebamipide micellar solution and plain Tween 20 solution.

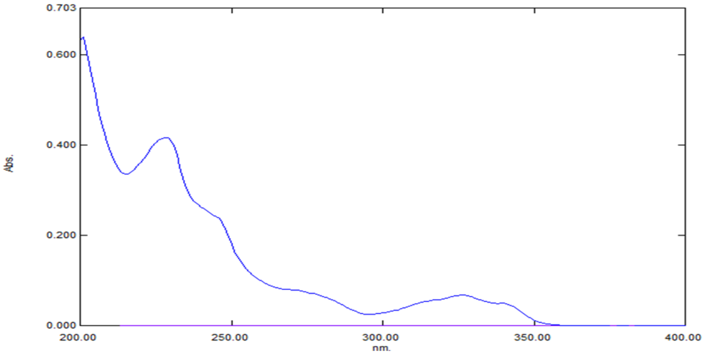

Figure S8. Overlay spectra of Rebamipide micellar solution and Pluronic F127 solution.

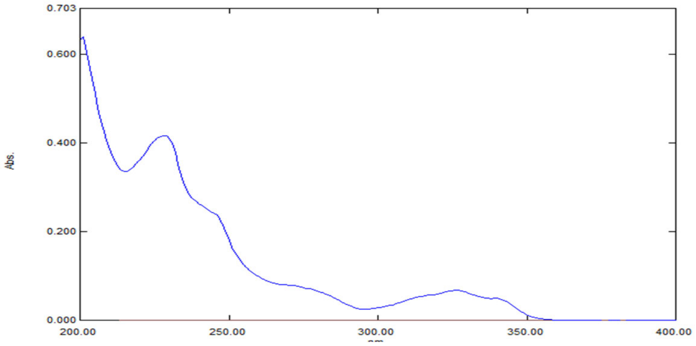

Figure S9. Overlay spectra of Rebamipide micellar solution and Pluronic F68 solution.

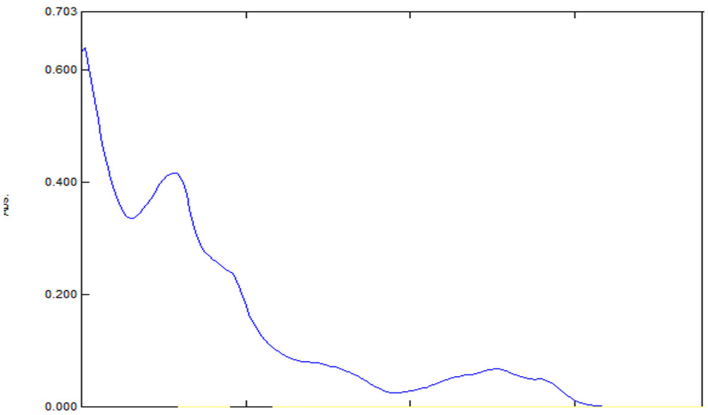

Figure S10. Overlay spectra of Rebamipide micellar solution and Labrasol solution.

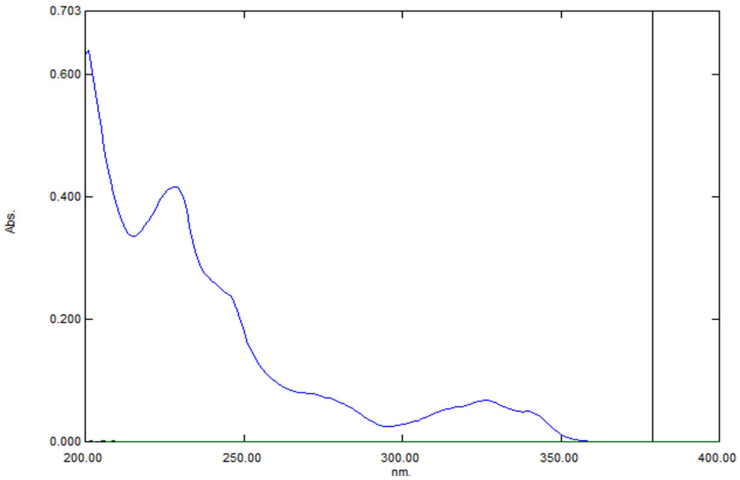

Figure S11. Overlay spectra of Rebamipide solution and Ocular insert extract after 24 hrs.

Table S4. DL and QL for REB.

Components	Methanol		STF	
	LOD (μg/ml)	LOQ (μg/ml)	LOD (μg/ml)	LOQ (μg/ml)
REB	0.140	0.424	0.095	0.289

Table S5. Saturation Solubility Data of Rebamipide in Different Surfactant Systems.

Formulation	Y1	Y2	Y3	Solubility (μg/mL ± SD)
Rebamipide (STF)	0.272	0.261	0.282	26.05 ± 1.00
Pluronic F127	0.444	0.446	0.447	212.85 ± 0.73
Pluronic F68	0.238	0.236	0.233	113.14 ± 1.19
Tween 80	0.552	0.549	0.554	263.18 ± 1.19
Tween 20	0.279	0.275	0.280	133.24 ± 1.26
Solutol HS 15	0.646	0.642	0.642	306.71 ± 1.10
Labrasol	0.356	0.358	0.358	170.91 ± 0.55

Table S6. Assay of Rebamipide-Loaded Ocular Inserts.

Formulation/System	Y1	Y2	Y3	Found (µg/mL ± SD)
Plain REB	0.222	0.251	0.278	21.41 ± 2.28
Pluronic F127	0.217	0.227	0.224	95.80 ± 2.09
Pluronic F68	0.194	0.202	0.210	87.40 ± 3.25
Tween 80	0.222	0.228	0.233	97.83 ± 2.24
Tween 20	0.210	0.215	0.219	92.55 ± 1.83
Solutol HS 15	0.215	0.218	0.223	94.17 ± 1.64
Labrasol	0.225	0.231	0.237	99.19 ± 2.44

Table S7. Drug Leaching During Sterilization of Rebamipide-Loaded Ocular Inserts.

Formulation/System	Y1	Y2	Y3	Drug Leached (µg/mL ± SD)
Plain REB	0.325	0.355	0.386	10.20 ± 0.87
Pluronic F127	0.557	0.601	0.646	17.21 ± 1.27
Pluronic F68	0.404	0.440	0.475	12.60 ± 1.01
Tween 80	0.598	0.650	0.702	18.59 ± 1.48
Tween 20	0.451	0.492	0.534	14.10 ± 1.18
Solutol HS 15	0.502	0.552	0.602	15.80 ± 1.42
Labrasol	0.630	0.693	0.757	19.83 ± 1.81

Table S8. UV–Visible Spectroscopic Absorbance Data (Y1) for Drug Release Study of Rebamipide-Loaded Ocular Inserts.

Time (h)	Solutol HS 15	Tween 80	Pluronic F127	Labrasol	Tween 20	Pluronic F68	Plain REB
0	0.00	0.00	0.00	0.00	0.00	0.00	0.00
0.5	0.125	0.085	0.081	0.047	0.035	0.017	0
1	0.256	0.223	0.162	0.108	0.073	0.056	0.141
2	0.432	0.319	0.225	0.197	0.131	0.075	0.175
3	0.393	0.318	0.251	0.169	0.111	0.071	0.145
4	0.429	0.322	0.247	0.181	0.122	0.072	0.085
5	0.413	0.325	0.224	0.175	0.126	0.081	0.026
6	0.2	0.236	0.165	0.134	0.078	0.034	
7	0.131	0.101	0.131	0.053	0.044	0.071	
8	0.137	0.138	0.127	0.117	0.077	0.111	
12	0.577	0.557	0.6	0.814	0.796	0.567	
24	0.203	0.264	0.341	0.366	0.436	0.474	

Table S9. UV–Visible Spectroscopic Absorbance Data (Y2) for Drug Release Study of Rebamipide-Loaded Ocular Inserts.

Time (h)	Solutol HS 15	Tween 80	Pluronic F127	Labrasol	Tween 20	Pluronic F68	Plain REB
0	0	0	0	0	0	0	0
0.5	0.13	0.105	0.098	0.066	0.044	0.019	0
1	0.297	0.24	0.17	0.117	0.087	0.067	0.147
2	0.42	0.32	0.234	0.198	0.131	0.08	0.175
3	0.416	0.341	0.249	0.176	0.128	0.073	0.145
4	0.421	0.32	0.249	0.186	0.12	0.076	0.087
5	0.413	0.333	0.247	0.164	0.109	0.087	0.026
6	0.2	0.224	0.178	0.137	0.088	0.03	
7	0.131	0.102	0.113	0.068	0.051	0.083	
8	0.145	0.125	0.113	0.121	0.08	0.117	
12	0.589	0.575	0.614	0.823	0.811	0.58	
24	0.204	0.266	0.353	0.372	0.437	0.481	

Table S10. UV–Visible Spectroscopic Absorbance Data (Y3) for Drug Release Study of Rebamipide-Loaded Ocular Inserts.

Time (h)	Solutol HS 15	Tween 80	Pluronic F127	Labrasol	Tween 20	Pluronic F68	Plain REB
0	0	0	0	0	0	0	0
0.5	0.127	0.118	0.109	0.075	0.047	0.02	0.001
1	0.317	0.247	0.181	0.123	0.097	0.077	0.15
2	0.421	0.321	0.233	0.201	0.132	0.08	0.175
3	0.413	0.336	0.25	0.169	0.131	0.075	0.146
4	0.424	0.325	0.247	0.197	0.117	0.08	0.085
5	0.412	0.336	0.244	0.16	0.113	0.087	0.026
6	0.205	0.233	0.184	0.135	0.088	0.03	
7	0.127	0.095	0.11	0.068	0.047	0.087	
8	0.157	0.117	0.116	0.134	0.081	0.112	
12	0.594	0.575	0.618	0.819	0.814	0.592	
24	0.199	0.26	0.351	0.378	0.438	0.482	

Table S11. UV–Visible Spectroscopic Absorbance Data (Y1) for Ex-vivo permeation Study of Rebamipide-Loaded Ocular Inserts.

Time (h)	Solutol HS 15	Tween 80	Pluronic F127	Labrasol	Tween 20	Pluronic F68	Plain REB
0	0	0	0	0	0	0	0
0.5	0.022	0.011	0.009	0	0	0	0
1	0.133	0.106	0.075	0.041	0.02	0	0
2	0.274	0.214	0.15	0.111	0.067	0.022	0.019
3	0.382	0.304	0.214	0.157	0.105	0.047	0.076
4	0.468	0.375	0.268	0.199	0.132	0.069	0.111
5	0.52	0.406	0.294	0.205	0.128	0.069	0.124
6	0.499	0.419	0.31	0.216	0.136	0.067	
7	0.448	0.378	0.292	0.2	0.129	0.087	
8	0.425	0.352	0.279	0.21	0.137	0.115	
12	0.572	0.49	0.444	0.471	0.408	0.298	
24	0.864	0.919	1.03	0.11	0.119	0.118	

Table S12. UV–Visible Spectroscopic Absorbance Data (Y2) for Ex-vivo permeation Study of Rebamipide-Loaded Ocular Inserts.

Time (h)	Solutol HS 15	Tween 80	Pluronic F127	Labrasol	Tween 20	Pluronic F68	Plain REB
0	0	0	0	0	0	0	0
0.5	0.024	0.013	0.008	0	0	0	0
1	0.137	0.107	0.075	0.043	0.023	0	0
2	0.28	0.218	0.154	0.117	0.07	0.025	0.021
3	0.392	0.23	0.224	0.165	0.111	0.05	0.077
4	0.481	0.398	0.28	0.206	0.137	0.074	0.097
5	0.532	0.432	0.302	0.211	0.133	0.08	0.087
6	0.509	0.44	0.315	0.226	0.14	0.07	
7	0.456	0.393	0.298	0.207	0.136	0.088	
8	0.425	0.364	0.286	0.215	0.141	0.12	
12	0.577	0.505	0.457	0.481	0.417	0.311	
24	0.864	0.934	0.104	0.112	0.121	0.119	

Table S13. UV–Visible Spectroscopic Absorbance Data (Y3) for Ex-vivo permeation Study of Rebamipide-Loaded Ocular Inserts.

Time (h)	Solutol HS 15	Tween 80	Pluronic F127	Labrasol	Tween 20	Pluronic F68	Plain REB
0	0	0	0	0	0	0	0
0.5	0.024	0.013	0.007	0	0	0	0
1	0.134	0.106	0.074	0.042	0.022	0	0
2	0.278	0.215	0.151	0.113	0.069	0.023	0.019
3	0.388	0.312	0.221	0.162	0.108	0.05	0.076
4	0.476	0.379	0.277	0.203	0.135	0.073	0.095
5	0.531	0.412	0.299	0.21	0.133	0.072	0.087
6	0.507	0.426	0.312	0.312	0.139	0.07	
7	0.456	0.384	0.297	0.188	0.134	0.089	
8	0.424	0.358	0.283	0.199	0.138	0.119	
12	0.574	0.497	0.453	0.466	0.414	0.307	
24	0.863	0.928	0.103	0.108	0.119	0.118	
